# The ARRE RING-Type E3 Ubiquitin Ligase Negatively Regulates Cuticular Wax Biosynthesis in *Arabidopsis thaliana* by Controlling ECERIFERUM1 and ECERIFERUM3 Protein Levels

**DOI:** 10.3389/fpls.2021.752309

**Published:** 2021-10-26

**Authors:** Shuang Liu, Meixuezi Tong, Lifang Zhao, Xin Li, Ljerka Kunst

**Affiliations:** ^1^Department of Botany, University of British Columbia, Vancouver, BC, Canada; ^2^Michael Smith Laboratories, University of British Columbia, Vancouver, BC, Canada

**Keywords:** Arabidopsis, cuticle, cuticular wax biosynthesis, AtARRE, E3 ligase, CER1, CER3

## Abstract

The outer epidermal cell walls of plant shoots are covered with a cuticle, a continuous lipid structure that provides protection from desiccation, UV light, pathogens, and insects. The cuticle is mostly composed of cutin and cuticular wax. Cuticular wax synthesis is synchronized with surface area expansion during plant development and is associated with plant responses to biotic and abiotic stresses. Cuticular wax deposition is tightly regulated by well-established transcriptional and post-transcriptional regulatory mechanisms, as well as post-translationally *via* the ubiquitin-26S proteasome system (UPS). The UPS is highly conserved in eukaryotes and involves the covalent attachment of polyubiquitin chains to the target protein by an E3 ligase, followed by the degradation of the modified protein by the 26S proteasome. A large number of E3 ligases are encoded in the Arabidopsis genome, but only a few have been implicated in the regulation of cuticular wax deposition. In this study, we have conducted an E3 ligase reverse genetic screen and identified a novel RING-type E3 ubiquitin ligase, AtARRE, which negatively regulates wax biosynthesis in Arabidopsis. Arabidopsis plants overexpressing AtARRE exhibit glossy stems and siliques, reduced fertility and fusion between aerial organs. Wax load and wax compositional analyses of AtARRE overexpressors showed that the alkane-forming branch of the wax biosynthetic pathway is affected. Co-expression of AtARRE and candidate target proteins involved in alkane formation in both *Nicotiana benthamiana* and stable Arabidopsis transgenic lines demonstrated that AtARRE controls the levels of wax biosynthetic enzymes ECERIFERUM1 (CER1) and ECERIFERUM3 (CER3). CER1 has also been confirmed to be a ubiquitination substrate of the AtARRE E3 ligase by an *in vivo* ubiquitination assay using a reconstituted *Escherichia coli* system. The *AtARRE* gene is expressed throughout the plant, with the highest expression detected in fully expanded rosette leaves and oldest stem internodes. *AtARRE* gene expression can also be induced by exposure to pathogens. These findings reveal that wax biosynthesis in mature plant tissues and in response to pathogen infection is controlled post-translationally.

## Introduction

The primary aerial surfaces of land plants are covered with a cuticle, a continuous lipidic layer that restricts transpirational water loss, reflects harmful UV light, and prevents organ fusions during development (Reicosky and Hanover, 1978; Sieber et al., 2000; Riederer and Schreiber, 2001; Riederer, 2006). The cuticle also serves as a protective barrier against pathogens and insects (Müller, 2018; Ziv et al., 2018) and is involved in drought-stress signaling (Wang et al., 2011).

The cuticle is mostly composed of cutin and cuticular wax (Samuels et al., 2008). Cutin is a polymer of oxidized 16- and 18-carbon (C16 and C18) fatty acids and glycerol (Beisson et al., 2012), which forms the structural scaffold of the cuticle. Cuticular wax embeds and overlays this cutin matrix and is composed of very long-chain fatty acids (VLCFAs; C20-C38) and their derivatives, including alkanes, aldehydes, primary and secondary alcohols, ketones, and esters. Small amounts of triterpenoids, flavonoids, or sterols may also be present (Jetter et al., 2006; Buschhaus and Jetter, 2011). Wax composition varies among plant species, as well as between different organs, tissues, and developmental stages of the same plant species. These variations in wax composition affect the biochemical and physical properties of the plant surface, which helps the plant adapt to different environments.

Cuticular wax is synthesized by epidermal cells. C16 and C18 fatty acids are made in the plastid and activated to acyl-CoA thioesters, which are translocated to the endoplasmic reticulum (ER) for further elongation to VLC acyl-CoA wax precursors by a fatty acid elongase (FAE) complex (Haslam and Kunst, 2013a). In addition, the ECERIFERUM2-LIKE (CER2-LIKE) family of proteins is required for the formation of C30 to C34 VLC acyl-CoAs (Haslam et al., 2017). Following elongation, VLC acyl-CoAs are modified by one of two pathways, either the acyl reduction pathway, which generates primary alcohols and wax esters, or the alkane-forming pathway, which produces aldehydes, alkanes, secondary alcohols, and ketones (Samuels et al., 2008). In Arabidopsis (*Arabidopsis thaliana*) leaves and stems, cuticular wax is predominantly derived from the alkane-forming pathway. As the major wax component, alkanes represent over 70 and 50% of the total wax load in leaves and stems, respectively (Bourdenx et al., 2011). It has been proposed that the formation of alkanes is catalyzed by a multiprotein complex comprising CER1, CER3, and a cytochrome B5 protein (CYTB5) that converts VLC acyl-CoAs to alkanes with aldehydes as intermediates (Rowland et al., 2007; Bourdenx et al., 2011; Bernard et al., 2012). CYTB5 isoforms interact with CER1 and provide the electron(s) required for this redox-dependent reaction. The CER1 and CER3 proteins are integral membrane proteins with 35% amino acid identity that contain eight conserved His clusters in their N-terminal domain and an uncharacterized WAX2 domain at their C-terminus (Aarts et al., 1995; Chen et al., 2003; Bernard et al., 2012). In Arabidopsis stems, alkanes can be further oxidized to secondary alcohols and ketones by a cytochrome P450 enzyme, the MID-CHAIN ALKANE HYDROXYLASE1 (MAH1; Greer et al., 2007).

Wax biosynthesis is tightly controlled throughout plant development and in response to biotic and abiotic stresses. Forward and reverse genetic studies in Arabidopsis, barley (*Hordeum vulgare*), maize (*Zea mays*), rice (*Oryza sativa*), and tomato (*Solanum lycopersicum*) have significantly improved our understanding of cuticular wax deposition and regulatory pathways controlling this process (Samuels et al., 2008; Yeats and Rose, 2013). Production of cuticular waxes is primarily under transcriptional regulation. Several independent studies have demonstrated that the WAX INDUCER1/SHINE1 (WIN1/SHN1) transcription factor, known to predominantly regulate cutin production, also indirectly affects wax synthesis (Aharoni et al., 2004; Broun et al., 2004; Kannangara et al., 2007). Other transcription factors, including MYB16, MYB30, MYB94, MYB96, MYB106, and WRINKLED4, have been reported to positively regulate wax synthesis in Arabidopsis stems and leaves (Raffaele et al., 2008; Seo et al., 2011; Oshima et al., 2013; Lee et al., 2014; Lee and Suh, 2015b; Park et al., 2016). Conversely, the DEWAX and DEWAX2 transcription factors act as repressors of wax production in Arabidopsis (Go et al., 2014; Kim et al., 2018).

In addition to the transcriptional regulation described above, characterization of the Arabidopsis *CER7* gene and suppressors of the *cer7* mutant resulted in the discovery of a post-transcriptional regulatory mechanism that affects stem wax deposition during inflorescence development. It involves CER7-mediated *CER3* gene silencing by trans-acting small interfering RNAs (tasiRNAs; Hooker et al., 2007; Lam et al., 2012, 2015). Recently, another type of small RNAs, microRNAs (miRNAs), were also shown to participate in the regulation of wax synthesis. Specifically, miR156 targets the SQUAMOSA PROMOTER BINDING PROTEIN-LIKE 9 (SPL9) transcription factor that positively regulates the expression of the alkane-forming enzyme CER1 through direct binding to the *CER1* promoter. Furthermore, SPL9 was shown to be involved in the optimization of diurnal wax production in Arabidopsis stems and leaves by direct protein–protein interaction with a negative regulator of wax synthesis DEWAX (Li et al., 2019).

Work by several groups demonstrated that wax biosynthesis in Arabidopsis is also post-translationally controlled by the ubiquitin-proteasome system (UPS). The UPS involves two distinct steps: the covalent attachment of a polyubiquitin chain consisting of at least four ubiquitin residues to the protein target, followed by the degradation of the modified protein by the 26S proteasome. Ubiquitination is catalyzed by three enzymes: a ubiquitin-activating enzyme (E1), a ubiquitin-conjugating enzyme (E2), and a ubiquitin ligase (E3). Among these proteins, E3 ligases play key roles in determining substrate specificity (Hershko and Ciechanover, 1998; Vierstra, 2009). Several Arabidopsis E3 ligases have been shown to be involved in regulating cuticular wax deposition. Characterization of the wax-deficient *cer9* mutant and isolation of the *CER9* gene revealed that it encodes a putative E3 ligase, although its enzyme activity and ubiquitination substrate have not been determined (Lü et al., 2012). More recently, MYB30-INTERACTING E3 LIGASE 1 (MIEL1) has been shown to negatively regulate cuticular wax biosynthesis in Arabidopsis stems by targeting MYB30 and MYB96 transcription factors for degradation (Lee and Seo, 2016; Gil et al., 2017). Additionally, F-box protein SAGL1 targets wax biosynthetic enzyme CER3 for degradation thereby negatively regulating cuticular wax production in response to changes in ambient humidity (Kim et al., 2019). In rice, the DROUGHT HYPERSENSITIVE E3 ligase negatively regulates wax production by targeting the RICE OUTERMOST CELL-SPECIFIC GENE4 (ROC4) transcription factor involved in drought-stress response for degradation by the UPS (Wang et al., 2018b).

Based on the presence of over 1,400 putative E3 ligases encoded in the Arabidopsis genome (Kraft et al., 2005) and their importance in the regulation of plant responses to environmental stress, we reasoned that additional E3 ligases may be involved in the control of cuticular wax deposition. Here, we report the identification of a RING-type E3 ubiquitin ligase named ABA-related RING-type E3 ligase (AtARRE) that negatively regulates wax production by promoting the degradation of wax biosynthetic enzymes CER1 and CER3. This E3 ligase was previously reported to be involved in abscisic acid (ABA) signaling, but its ubiquitination target has not been identified (Wang et al., 2018a). Our results demonstrate that Arabidopsis plants overexpressing AtARRE display glossy stems and siliques, markedly reduced wax loads, and often aerial organ fusions and reduced fertility. Co-expression of AtARRE and candidate substrates in both *Nicotiana benthamiana* and stable Arabidopsis transgenic lines indicates that CER1 and CER3 wax biosynthetic enzymes are targeted by the AtARRE for degradation *via* the 26S proteasome. The *AtARRE* gene is highly expressed in tissues that exhibit no or low wax production, such as roots and cotyledons in older developing seedlings, as well as fully expanded rosette leaves and older internodes at the bottom of the stem in mature plants. *AtARRE* expression can also be induced by pathogen infection. Taken together, our results suggest that AtARRE acts as a quick and efficient switch for turning off wax biosynthesis in tissues where it is no longer needed and upon exposure to pathogens.

## Materials and Methods

### Plant Material and Growth Conditions

*Arabidopsis thaliana* ecotype Columbia-0 (Col-0) wild type was used in this study. Arabidopsis T-DNA insertion lines *atarre-1* (SALK_094303), *atarre-2* (SALK_034426C; Alonso et al., 2003), and the *cer1-4* and *cer4-4* mutants were obtained from the Arabidopsis Biological Resource Center (ABRC).^1^ GABI-KAT T-DNA line *atarre-3* (GABI_383G01) was obtained from gabi-kat.de (Kleinboelting et al., 2012). *cer3-6* was a gift from Dr. Takuji Wada (RIKEN, Japan). AtARRE overexpression lines in Col-0 background were identified from the *snc1*-influencing plant E3 ligase reverse (SNIPER) genetic screen (Tong et al., 2017).

Arabidopsis seeds were germinated on *Arabidopsis thaliana* (AT) medium (Haughn and Somerville, 1986) supplemented with 1% (w/v) agar and appropriate antibiotics for transgene selection. Seven-day-old seedlings were transplanted to soil (Sunshine Mix 4 or 5, SunGro, Canada) supplemented with liquid AT medium and grown in a plant growth chamber at 20°C under continuous light [100μmolm^−2^ s^−1^ of photosynthetically active radiation (PAR)]. Arabidopsis seeds grown for Agrobacterium-mediated transformation were directly spread on the soil supplemented with liquid AT medium at a density of 100 seeds/6″ pot and grown as described above.

*Nicotiana benthamiana* seeds were sown directly on soil (Sunshine Mix 4 or 5, SunGro, Canada) supplemented with liquid AT medium at a density of 1 seed/3.5″ square pot. Plants were grown under a 14-h light (25°C with 100μmolm^−2^ s^−1^ PAR) and 10-h dark (20°C) cycle. For the transient expression assay, 4- to 5-week-old plants were taken out of the growth chamber and left at room temperature for 3 to 4h before infiltration.

### RNA Isolation, RT-PCR, and qPCR

Plant tissues were collected and immediately frozen in liquid nitrogen. RNA was extracted from Arabidopsis leaves, stems, flowers, seedlings, and roots using TRIzol (Thermo Fisher Scientific) according to the manufacturer’s protocol. RNA was isolated from Arabidopsis siliques using a phenol:chloroform:isoamyl extraction and precipitated by lithium chloride and sodium acetate (Wilkins and Smart, 1996). RNA integrity was examined on a 1% standard agarose gel, and RNA was quantified using a NanoDrop 8000 (Thermo Scientific). Genomic DNA was removed by DNase I treatment (New England Biolabs) following the manufacturer’s protocol, and single-stranded cDNA was synthesized from equal amounts of purified RNA using iScript RT Supermix (Bio-Rad). *ACTIN1* was used as an internal control. The iQ SYBR Green Supermix (Bio-Rad) was used in 20μl reactions to perform qPCR in an iQ5 Multicolor Real-Time PCR Detection System (Bio-Rad) as specified by the manufacturer. Four technical replicates were performed for each sample, and gene expression levels were analyzed using the Pfaffl method (Pfaffl, 2001).

### Cloning of Genes in Plant Expression Vectors

Standard methods were used for cloning, and all primer sequences are given in Supplementary Table 1. All constructs were confirmed by sequencing.

The *pGreenST/35S:HA-AtARRE* construct, which was prepared for the SNIPER screen (Tong et al., 2017), was used as the site-directed mutagenesis template. Of the five splice variants known for the *AtARRE* gene (AT5G66070), AT5G66070.1 was used for the work described here. The *35S:HA-AtARRE^(H197Y,H200Y)^* site-directed mutagenesis construct was generated using primers H197200Y_F and H197200Y_R designed using the one-step site-directed mutagenesis method (Zheng et al., 2004). The PCR amplification was carried out using Phusion High Fidelity Polymerase (Thermo Fisher Scientific). The PCR products were separated by gel electrophoresis, purified using a PCR Purification Kit (BioBasic), and were further treated with restriction enzyme DpnI (New England Biolabs). The mutations in the *35S:HA-AtARRE^(H197Y,H200Y)^* construct were confirmed by sequencing.

The *35S:GFP-CER3* construct was prepared using Gateway cloning (Thermo Fisher) and destination vectors from Nakagawa et al. (2007). The coding sequence of the *CER3* gene was amplified from WT cDNA using CER3cDNA_attbF and CER3cDNA_attbR_WSTOP primers and recombined into the entry vector pDONR221. The insert was then transferred into the destination vector pGWB6 to generate pGWB6/*35S:GFP-CER3* and into pGWB15 to generate pGWB15/*35S:HA-CER3*. The *CER3* coding sequence without stop codon was also amplified using CER3cDNA_attbF and CER3cDNA_attbR_NoSTOP primers, recombined to pDONR221, and then transferred to the destination vector pGWB5 to generate pGWB5/*35S:CER3-GFP*.

The *35S:CER1-GFP* construct was made and provided by Dr. Hugo Zheng (McGill University, Canada). The coding region of the *CER1* gene was subcloned into the vector *pVKH18/35S:GFPC* (Dean et al., 2007) to produce the C-terminal CER1-GFP fusion under the control of the enhanced *35S* promoter. The *35S* promoter fragment was then removed from the vector *pVKH18/35S:CER1-GFP* and replaced with the *CER6* promoter using HindIII and XbaI to generate *pVKH18/CER6p:CER1-GFP*. The construct *pBIN/35S:HDEL-mCherry* was provided by Dr. Mathias Schuetz (Nelson et al., 2007), and the construct *pGreenST/35S:HA-SNIPER2* was described previously (Wu et al., 2020).

To generate the construct *AtARREp:GUS*, a fragment of 808bp immediately upstream of the putative AtARRE start codon, which includes the 5′ UTR of *AtARRE*, as well as 3′ UTR and the last intron of the previous gene, was amplified from WT genomic DNA using LP_attb1_AtARRE and RP1_attb2_AtARRE and recombined into pDONR221 before being introduced into pGWB3 (Nakagawa et al., 2007; Vincent et al., 2018) using GATEWAY cloning (Thermo Fisher).

### Cloning of Genes in Bacterial Expression Vectors

Standard methods were used for cloning, and all primer sequences are given in Supplementary Table 1. All constructs were confirmed by sequencing.

To generate the construct *pET28b/AtARRE-HIS* for the *in vitro* ubiquitination assay, a 326bp fragment of coding sequence downstream of the transmembrane domains and upstream of the stop codon of *AtARRE* was amplified from WT cDNA using AtARRETMdel_F_EcoRI_28b and AtARRETMdel_R_SalI. This PCR product was ligated into the *pET28b* vector using EcoRI and SalI restriction sites to generate *pET28b/AtARRE-HIS*.

To reconstitute the plant ubiquitination cascade in *Escherichia coli*, Duet expression vectors (kindly provided by Dr. Dongping Lu, Chinese Academy of Science, China) *pCDFDuet/MBP-ABI3-HA-AtUBA1-S*, *pCDFDuet/AtUBA1-S*, *pACYCDue/AIP2-Myc-UBC8-S*, and *pET28a/FLAG-UBQ* were used to generate target co-expression constructs (Han et al., 2017). A 326bp fragment of coding sequence downstream of the transmembrane domains and upstream of the stop codon of *AtARRE* was amplified from WT cDNA and ligated into the BamHI and StuI-digested *pACYCDue/AIP2-Myc-UBC8-S* vector to generate *pACYCDue/AtARRE-Myc-UBC8-S*. An 837bp fragment of coding sequence downstream of the transmembrane domains and upstream of the stop codon of *CER1* was amplified from WT cDNA was ligated into the EcoRI and StuI-digested *pCDFDuet/MBP-ABI3-HA-AtUBA1-S* vector to generate *pCDFDuet/MBP-CER1-HA-AtUBA1-S*.

### Agrobacterium-Mediated Plant Transformation

To produce transgenic lines for E3 ubiquitin ligase activity test, degradation assay, and GUS assay, *35S:HA-AtARRE^(H197Y,H200Y)^*, *CER6p:CER1-GFP*, *AtARREp:GUS*, and *AtARREp:AtARRE-GUS* were introduced into *Agrobacterium tumefaciens* GV3101 cells carrying the pMP90 Ti plasmid. The pGreenST plasmid *35S:HA-AtARRE^(H197Y,H200Y)^* was co-transformed with the helper plasmid pSOUP (Hellens et al., 2000). Transformation of WT or *cer1-4* plants was carried out using the floral spray method (Chung et al., 2000). T1 transgenic seeds were harvested and screened on AT medium supplemented with 1% (w/v) agar and appropriate antibiotics.

### Transient Expression in *Nicotiana benthamiana*

Transient expression in *N. benthamiana* was carried out using 4- to 5-week-old plants. Agrobacterium cultures were grown overnight in 3ml of LB medium under antibiotic selection and diluted 1/20 in LB medium with antibiotics and 50μm acetosyringone and incubated for a further 3–5h. During this time, plants were taken out of the growth chamber and left at room temperature before infiltration. Cultures were centrifuged and resuspended in resuspension medium (4.43g/L MS, 10mm MES, and 150μm acetosyringone) at an optical density of 0.6 at A_600_. For co-expression of multiple constructs, suspensions were mixed in equal ratios. Agrobacterium suspension mixtures were infiltrated using a 1-ml syringe into the abaxial side of the *N. benthamiana* leaves. A permanent marker was used to mark the infiltrated area on the leaf. Infiltrated plants were incubated at room temperature for 48h, and then, leaf samples were collected for microscopic imaging and/or protein extraction.

### Cuticular Wax Extraction and Analysis by GC-FID

Cuticular wax extraction was performed using the method described by Haslam and Kunst (2013b). Briefly, the top 10cm of 4- to 6-week-old inflorescence stems were cut and photographed to allow stem surface area to be calculated by measuring the number of pixels of the two-dimensional area in Photoshop (Adobe), converting the values to cm^2^, and multiplying by π. After imaging, stems were submerged for 30s in chloroform containing 10μg tetracosane as an internal standard. After wax extraction, chloroform was evaporated under a stream of nitrogen gas and wax components were silylated in 10μlN, *O*-Bis (trimethylsilyl) trifluoroacetamide (BSTFA; Sigma), and 10μl pyridine for 1h at 80°C. After derivatization, the solvent was evaporated under nitrogen gas and waxes were re-dissolved in 30μl of chloroform for GC analysis. Samples were analyzed on an Agilent 7890A gas chromatograph equipped with a flame ionization detector (GC-FID) using an HP1 column (Agilent) in a 2.7:1 split mode with H_2_ as the carrier gas at a flow rate of 30ml/min. The gas chromatography program used was as follows: oven temperature was set at 50°C for 2min, raised by 40°C/min to 200°C and held for 1min, and then raised by 3°C/min to 320°C and held for 15min. Wax components were identified by comparing their retention times with those of the internal standards. Four biological replicates were processed for each line.

### Microscopy

Fluorescence signals of transiently expressed constructs in *N. benthamiana* were detected using a Perkin Elmer Ultraview VoX Spinning Disk Confocal Microscope. *N. benthamiana* leaf discs were mounted in distilled water and immediately imaged using a glycerol immersion lens. GFP was excited using a 488nm laser with an 515/30nm emission filter, and mCherry was excited using a 561nm laser with an 595/50nm emission filter. Confocal images were processed using the Volocity software (Perkin Elmer). GFP signal in infiltrated *N. benthamiana* leaf was also observed using a Nikon Eclipse 80i Scanning Laser Confocal Microscope excited with a 488nm laser with an 515/30nm emission filter.

For scanning electron microscopy (SEM), segments from the apical 1cm of dry stems were mounted onto stubs and sputter-coated with gold particles for 10min at 40mA in an SEM Prep 2 sputter coater (Nanotech). The coated samples were viewed using an S4700 field emission SEM (Hitachi) with an accelerating voltage of 5kV and a working distance of 12mm.

### GUS Histochemical Assay

Tissues at different developmental stages from transgenic lines expressing *AtARREp:GUS* constructs were immersed in GUS staining buffer {100mM Na-phosphate, 10mM EDTA, pH 7.0, 0.5mM K_3_[Fe(CN)_6_], 0.5mM K_4_[Fe(CN)_6_], 0.1% (v/v) Triton X-100, and 1mM 5-bromo-4-chloro-3-indolyl-β-D-glucuronide (X-gluc)} and incubated for 1 to 3h or overnight. The reaction was stopped by removing the GUS buffer and adding the 70% (v/v) ethanol. Chlorophyll was removed by incubating samples in 70–90% (v/v) ethanol before samples were examined under a Nikon SMZ18 Digital Microscope (Nikon, Japan).

### Protein Extraction and Immunoblotting

Plant tissues were ground in liquid nitrogen, and total proteins were extracted in buffer containing 50mM Tris-HCl, pH 7.5, 150mM NaCl, 1mM EDTA, 10% (v/v) glycerol, 1% Triton X-100, 1mM PMSF, and 1X Halt^™^ protease inhibitor cocktail (Thermo Fisher Scientific). After centrifugation at 18,000g for 20min at 4°C, the supernatant was transferred to a new tube and the concentration of protein extract was determined using the Bradford reagent (Bio-Rad).

For SDS-polyacrylamide gel electrophoresis (SDS-PAGE), 4X SDS loading buffer (200mM Tris-HCl, pH 6.8, 8% (w/v) SDS, 0.4% (w/v) bromophenol blue, 40% glycerol, and 400mM DTT) was added to solubilized protein samples, and 10–35μl of each protein sample was separated on a 10% acrylamide gel with 1% SDS at 200V constant voltage for 50–60min before being transferred to nitrocellulose membrane using a semi-dry blotting system (Bio-Rad) with Bjerrum Schafer-Nielsen buffer (Bio-Rad). Transfer was carried out at a constant voltage of 15V for 50min before the membrane was stained with Ponceau S, imaged, washed, and then blocked with 5% skim milk powder in Tris-buffered saline with 0.1% tween 20 (TBS-T). For immunoblotting, membranes were incubated with primary antibody for 1h at room temperature. Primary antibodies used were anti-GFP (dilution 1:5,000; mouse IgG; Roche), anti-HA (dilution 1:2,500; rat IgG; Roche), anti-HIS (dilution 1:1,000; mouse IgG; Santa Cruz Biotechnology), anti-FLAG (dilution 1:5,000; mouse IgG; Sigma), anti-GST (dilution 1:1,000; rabbit IgG; Sigma), anti-Myc (dilution 1:1,000; Invitrogen), and anti-Ub (dilution 1:1,000; mouse IgG; Sigma). Membranes were then washed three times for 10min each wash with TBS-T and then incubated with appropriate secondary antibodies, including anti-rabbit (dilution 1:10,000; Santa Cruz Biotechnology), anti-mouse (dilution 1:25,000; Santa Cruz Biotechnology), and anti-rat (dilution 1:10,000; Santa Cruz Biotechnology), for 1h at room temperature. The membrane was washed three times as above with TBS-T before horseradish peroxidase was detected with the ECL Prime western blotting detection kit (GE).

### Cell-Free Degradation Assay

Plant-derived protein degradation assays were performed as described in Wang et al. (2009), with modifications as follows. Total proteins were extracted from 8-day-old *CER6pro:CER1-GFP/cer1-4* transgenic seedlings and quantified. 80μl protein extracts were then incubated with or without 40μM MG132 (Sigma) at 30°C. Samples were taken at select time points, and the reaction was stopped by adding 5μl 4X SDS loading buffer. CER1-GFP protein abundance in each sample was determined by immunoblotting using anti-GFP antibody.

### *In vitro* Ubiquitination Assay

*In vitro* ubiquitination assays were performed as described in Zhao et al. (2013), with modifications as follows. The plasmid *pET28b/AtARRE-HIS* was transformed into *E. coli* strain Rosetta^™^2 (DE3) for protein production. A 500ml culture was grown in Terrific Broth (TB) medium [1.2% (w/v) tryptone, 2.4% (w/v) yeast extract, 0.4% (v/v) glycerol, 100mM K-PO_4_] until the exponential phase (OD_600_ =0.6–1.0) before protein production was induced by adding 0.5mM Isopropyl β-D-1-thiogalactopyranoside (IPTG). After growth overnight at 16–18°C, cells were collected by centrifugation at 6,000g for 5min and frozen in liquid nitrogen and stored at −80°C. Lysis buffer [50mM NaPO_4_, pH 7.5, 200mM NaCl, 0.1% (v/v) Triton X, 5% glycerol, 1mM PMSF, 1X Halt^™^ protease inhibitor cocktail, and 1mg/ml lysozyme] was added to the frozen sample pellets, thawed at 37°C for 1min, and resuspended. Lysate was cleared by centrifugation at 14,000g for 15min at 4°C followed by filtration through a 0.45 μm filter. The AtARRE-HIS recombinant proteins were purified using HisPur Ni-NTA Resin (Thermo Fisher) according to the manufacturer’s protocol. Purified recombinant proteins AtUBA2-His and GST-AtUBC8 were kindly provided by Dr. Oliver Xiao'ou Dong (Dong et al. 2018).

### *In vivo* Ubiquitination Assay in Bacteria

*In vivo* ubiquitination assays in bacteria were carried out using the system described by Han et al. (2017). *E. coli* strain BL21 (DE3) containing different combinations of the expression vectors were grown in 2ml of TB liquid medium with appropriate antibiotics at 37°C. When the culture A_600_ nm reached 0.4–0.6, 0.5mM IPTG was added to induce the recombinant protein expression. After induction, bacteria were further grown at 28°C for 10–12h, stored at 4°C overnight, and then harvested from 300μl of culture by centrifugation at 12,000g for 5min. The pellets were resuspended in 100μl 1x SDS loading buffer and boiled at 95°C for 5min followed by immunoblotting.

## Results

### Overexpression of *AtARRE* Results in Reduced Wax Accumulation on Arabidopsis Stems and Leaves

**Figure 1:**
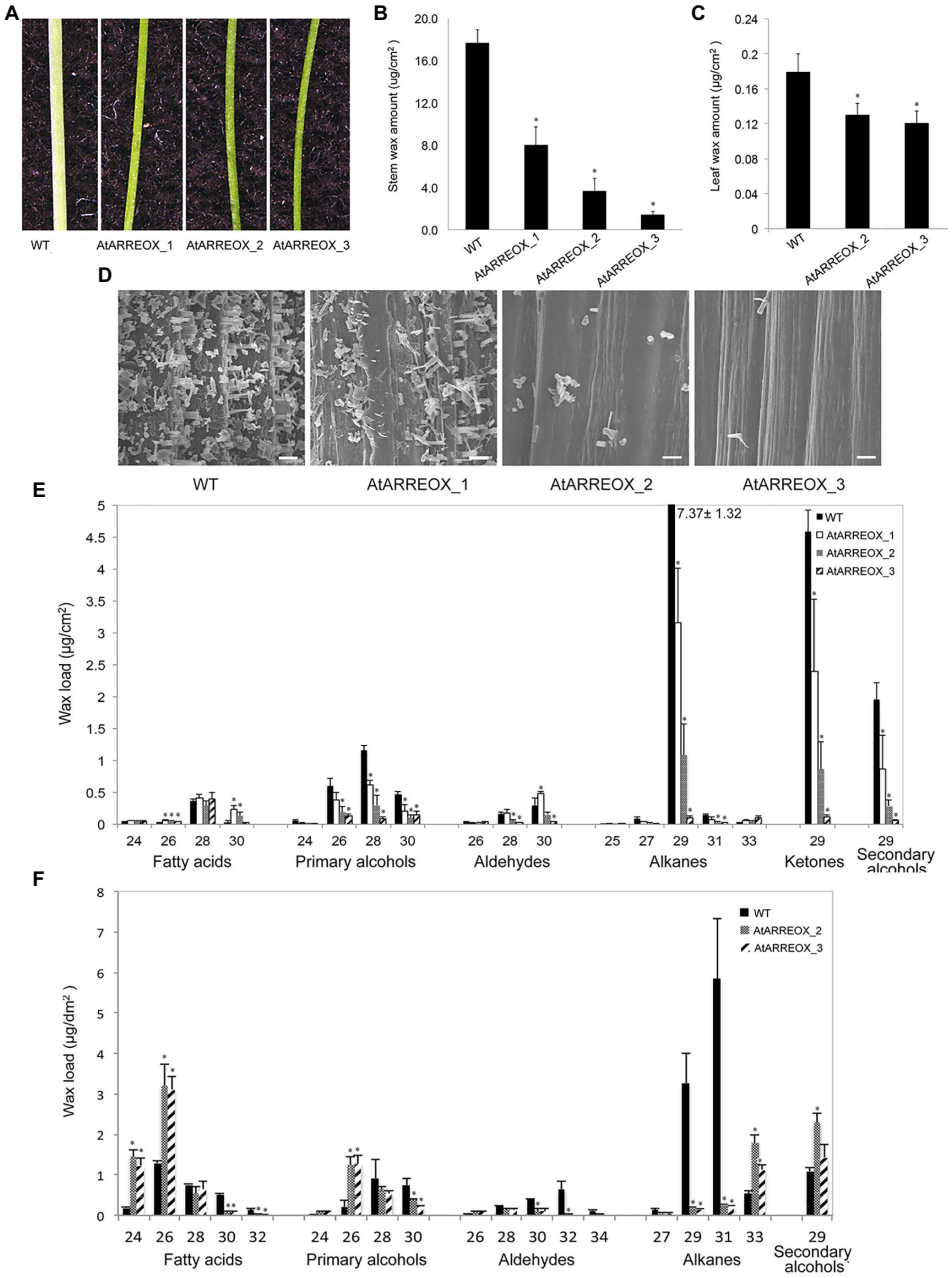
Characterization of the wax-deficient phenotypes of AtARREOX lines. **(A)** Stems of 6-week-old wild type (WT) and three representative AtARREOX lines. Total stem **(B)** and leaf **(C)** wax load of WT and AtARREOX lines as determined by GC-FID. Error bars represent means±SD (*n*=4). **(D)** SEM images of WT and AtARREOX inflorescence stem surfaces. Scale bar=20μm. The stem **(E)** and rosette leaf **(F)** wax composition of WT and AtARREOX lines as measured by GC-FID. Error bars represent means±SD (*n*=4). Statistically significant differences between the WT and each AtARREOX line were determined by Student’s *t* test and are indicated by asterisks (*p*<0.05).

To identify novel E3 ligases involved in plant immunity, a SNIPER genetic screen has been carried out (Tong et al., 2017). In this screen, E3-ligase encoding genes induced during plant defense were overexpressed in the wild-type background. Unexpectedly, a number of independent transgenic plants with glossy bright green stems were uncovered among the T1 progeny, suggestive of altered cuticular wax accumulation (Figure 1A). In these plants, the *AtARRE/At5g66070* gene encoding a RING-type E3 ubiquitin ligase was expressed under the control of the cauliflower mosaic virus (CaMV) 35S promoter (Supplementary Figures S1A,B). Wax analysis of three representative AtARRE overexpression (AtARREOX) lines by gas chromatography demonstrated that they accumulated only 10–50% of the WT inflorescence stem wax and only ~65% of the WT leaf wax (Figures 1B,C). The stem wax phenotype was further evaluated by scanning electron microscopy (SEM); wild-type stem surface was densely and uniformly covered with column-, vertical plate-, and rod-shaped wax crystals, whereas AtARREOX lines displayed considerably lower density of all types of wax structures (Figure 1D).

The wax deficiency uncovered in the AtARREOX lines prompted us to examine the wax phenotypes of *atarre* mutants. We obtained three T-DNA insertion lines of *AtARRE* in the Col-0 ecotype and determined *AtARRE* gene expression in mutant alleles by qPCR. Even though we detected reduced levels of *AtARRE* transcript in all three T-DNA lines (Supplementary Figures S1A,B), we found no major differences in the total stem wax load or composition with respect to the wild type (Supplementary Figures S1C,D).

### AtARREOX Lines Exhibit Altered Wax Composition and Abnormal Organ Morphogenesis

To further investigate the role of AtARRE in cuticular wax biosynthesis, we carried out a detailed analysis of wax composition of AtARREOX lines. We found that amounts of all stem wax components were altered in AtARREOX plants in comparison with the wild type, and detected considerable changes in their relative proportions. In particular, there was a prominent decrease in absolute amounts of alkane pathway-derived compounds that could be attributed primarily to C29 alkanes (68–98.7% decrease), C29 ketones (50–97.6% decrease), and C29 secondary alcohols (56–97% decrease). Conversely, the amounts of fatty acids on AtARREOX stems were higher than in the wild type (115–167% increase) and so was the relative proportion of fatty acids, aldehydes, and primary alcohols (Figure 1E, Supplementary Figure S1E). We also examined the wax composition of AtARREOX rosette leaves. As observed with stem wax, leaf wax also contained significantly reduced amounts of C29 and C31 alkanes relative to the wild type, but also increased amounts of C33 alkanes (Figure 1F). In addition, we detected a major increase in C24 and C26 fatty acids, as well as C26 primary alcohols (Figure 1F). Collectively, these data suggest that wax production by the alkane-forming pathway is impaired in AtARRE overexpressors.

**Figure 2:**
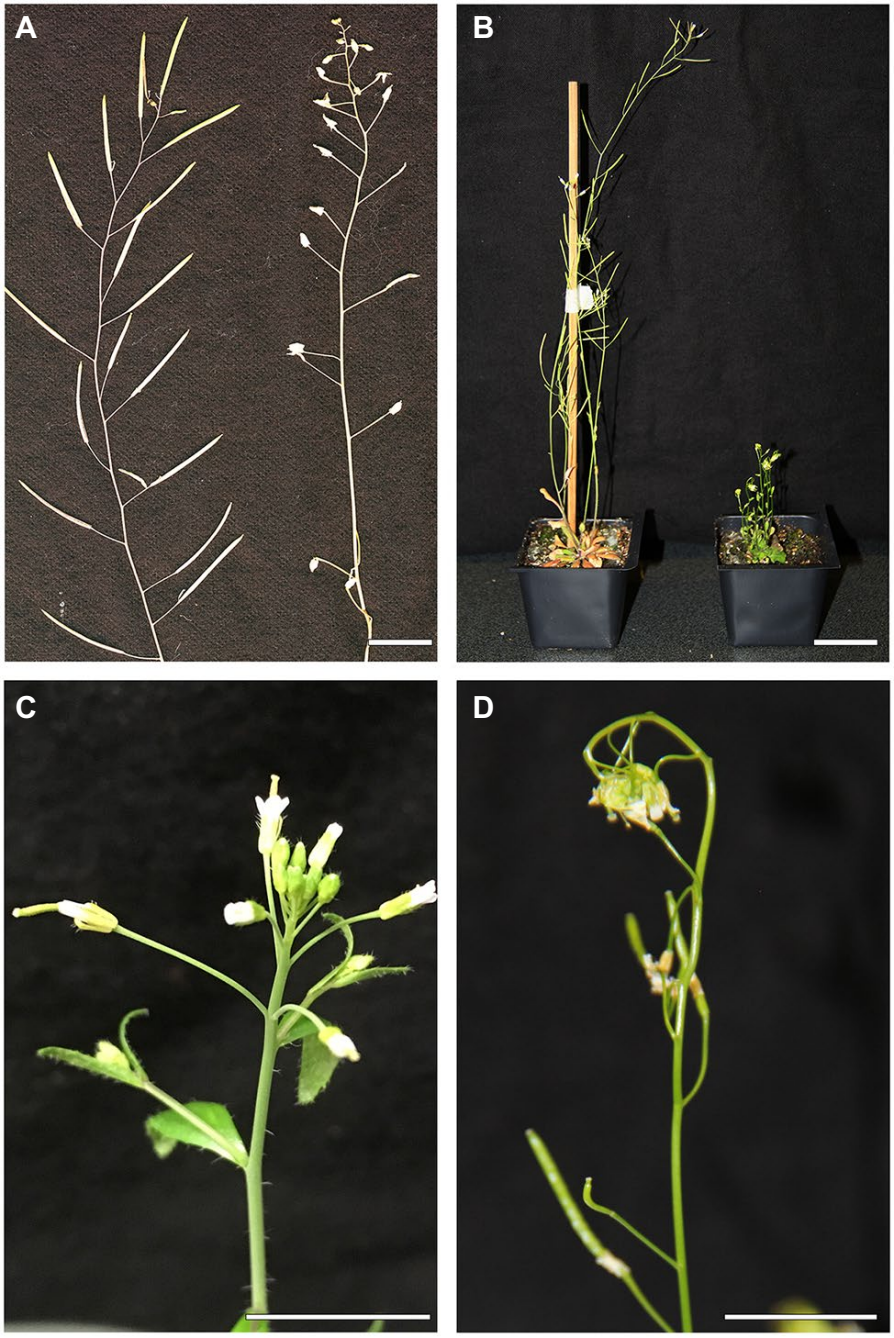
Abnormal organ morphogenesis in the AtARREOX lines. **(A)** Dry stems of WT (left) and a T3 AtARREOX_2 individual (right). **(B)** 6-week-old WT plant (left) and a T3 AtARREOX_3 plant (right). **(C)** Flowers of WT (Col-0). **(D)** Organ fusions between flower buds of a T3 AtARREOX_3 individual. Scale bars=2cm.

AtARREOX lines with the most severe wax deficiency displayed additional phenotypes including abnormal organ morphogenesis, dwarfism, organ fusions, and reduced fertility over multiple generations (Figure 2). For example, the inflorescence stems of AtARRE overexpressors were considerably shorter than the wild type. Additionally, these plants also exhibited organ fusions between flower buds, flowers, and siliques, as well as between flowers and leaves (Figures 2C,D). Reduced fertility was also often detected. In most cases, fertility could be restored by growing plants under high humidity, except in individuals with severe floral organ fusions (Supplementary Figure S2). Similar phenotypes have previously been reported for several Arabidopsis wax-deficient *eceriferum* mutants (Koornneef et al., 1989).

### AtARRE E3 Ubiquitin Ligase Activity Is Required for Its Function in Wax Deposition

**Figure 3:**
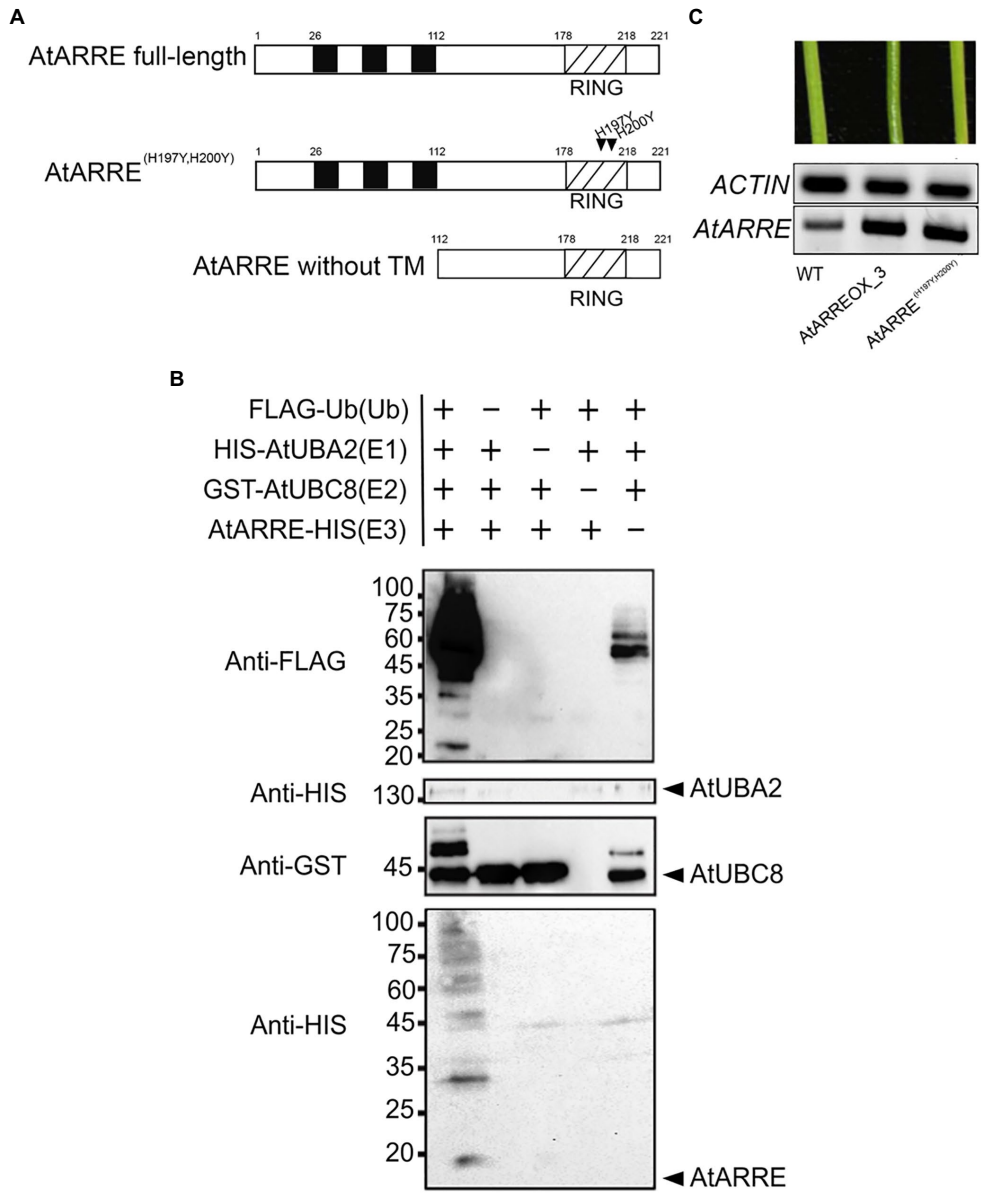
AtARRE exhibits E3 ubiquitin ligase activity. **(A)** Schematic representations of the full-length AtARRE protein (top), AtARRE protein with mutated RING domain (middle) and truncated AtARRE protein without transmembrane domains used for self-ubiquitination assay (bottom). Arrows show the point mutations in the RING domain. **(B)** *In vitro* ubiquitination assays were performed in the presence (+) or absence (−) of AtUBA2 (E1; 140kDa), AtUBC8 (E2; 44kDa), AtARRE (E3; 14.4kDa), and ubiquitin (Ub; 9.5kDa). Ubiquitination of AtARRE was detected by immunoblotting using an anti-FLAG antibody or anti-HIS antibody. AtUBC8 and AtUBA2 were detected by immunoblotting using anti-GST and anti-HIS antibody, respectively. Molecular mass markers are indicated on the left. **(C)** Stems of 6-week-old WT, AtARREOX_3 (T3 generation), and representative AtARRE^(H197YH200Y)^. *AtARRE* transcript accumulation in each sample was measured by RT-PCR. *ACTIN* was used as an internal control.

*AtARRE* (*AT5G66070*) encodes a predicted polypeptide of 221 amino acids with a molecular mass of 27kDa containing three transmembrane domains located at the N-terminus and a RING domain located at the C-terminus (Figure 3A). RING domain proteins act as E3 ligases by binding to an E2-ubiquitin thioester and catalyzing ubiquitin transfer (Deshaies and Joazeiro, 2009). Whether the RING domain-related E3 ubiquitin ligase activity of the AtARRE protein is required for its function in cuticular wax metabolism is not known. RING domain E3 ligases are known to undergo self-ubiquitination in the absence of their native substrate (Lorick et al., 1999). We used this feature of RING E3 ligases to determine whether AtARRE has E3 ligase activity. Unfortunately, the insolubility of the full-length AtARRE protein upon expression in *E. coli* prohibited purification of enough protein for the self-ubiquitination assay. Therefore, an AtARRE protein fragment without the N-terminal transmembrane domains was used to produce the recombinant AtARRE-HIS protein. Incubation of the purified AtARRE-HIS recombinant protein with E1, E2, ubiquitin, and ATP resulted in a laddering pattern characteristic of ubiquitination on a protein blot when anti-HIS antibodies were used for AtARRE-HIS detection. This laddering is indicative of a range of molecular weights for AtARRE-HIS as it carries ubiquitin chains of different lengths (Figure 3B). Such a laddering pattern was also detected when anti-Flag antibodies that were used for FLAG-Ub detection of Ub chains bound to AtARRE-HIS. Thus, AtARRE exhibits E3 ligase activity and undergoes self-ubiquitination *in vitro*. The self-ubiquitination of AtARRE was not observed when E1, E2, or ubiquitin were omitted from the assays.

Conserved Cys and His residues in the RING domain are critical for the E3 ligase activity (Deshaies and Joazeiro, 2009). Their substitution disrupts the RING domain and results in a dominant-negative form of E3 ligase predicted to confer the same phenotype as the loss of E3 ligase function. To further verify whether AtARRE E3 ligase activity is required for its function, we replaced the conserved AtARRE RING domain His-197 and His-200 with Tyr residues by site-directed mutagenesis and expressed the modified protein in wild-type plants. In contrast to AtARRE overexpression which caused wax deficiency, overexpression of the double mutant AtARRE^(H197YH200Y)^ protein had no effect on the stem wax load (Figure 3C). Thus, the E3 ligase activity of AtARRE is required for its function in stem wax deposition.

### AtARRE Overexpression Phenotypes Mimic *cer1* and *cer3* Wax-Deficient Mutants

**Figure 4:**
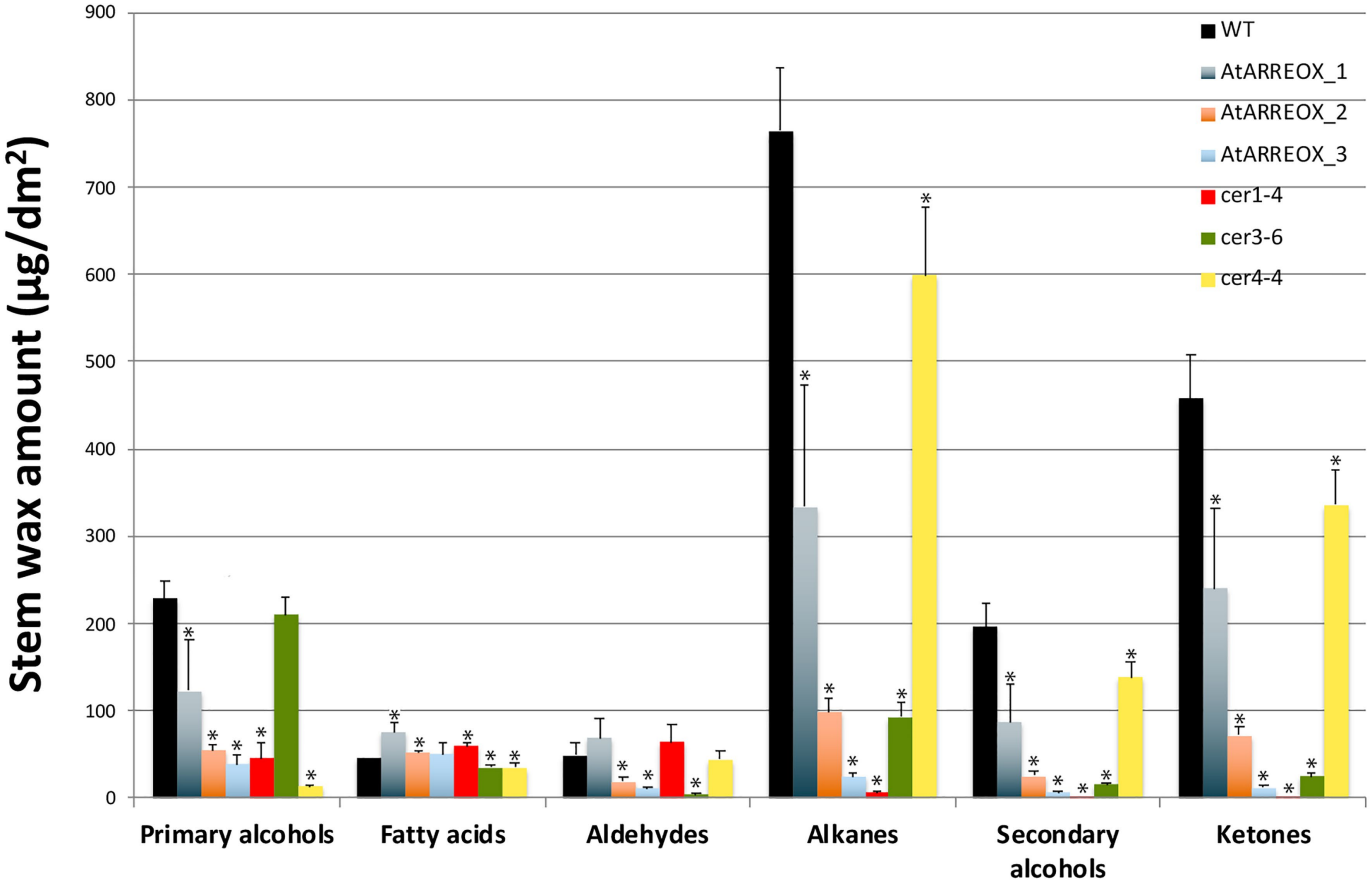
A comparison of stem wax load and composition of AtARRE overexpression lines, and *cer1-4*, *cer3-6*, and *cer4-4* mutants. Stem wax load and composition of 6-week-old WT, AtARREOX lines, *cer1-4*, *cer3-6*, and *cer4-4* were determined by GC-FID. Values are means of four biological replicates, and error bars represent SD. Statistically significant differences of wax component amounts between the WT and different genotypes (*p*<0.05) were determined by Student’s *t* test and are indicated by asterisks.

E3 ligase-mediated ubiquitination of proteins in most cases results in their degradation by the 26S proteasome. Because the most conspicuous result of AtARRE overexpression was reduced cuticular wax accumulation on Arabidopsis inflorescence stems, we hypothesized that AtARRE may act as a negative regulator of wax deposition by ubiquitinating, and thus targeting for degradation, a key player involved in wax biosynthesis. If this is the case, identifying the ubiquitination substrate of AtARRE is critical for determining its biological function. As a first step in uncovering potential candidate ubiquitination substrates, we compared stem wax compositional changes of AtARRE overexpressors with those of *ecerifierum* (*cer*) Arabidopsis mutants caused by loss-of-function mutations in wax biosynthetic genes (Figure 4, Supplementary Figure S3). AtARRE overexpressors displayed dramatically reduced alkane, secondary alcohols, and ketone levels on their stem surfaces, similar to null mutants disrupted in *CER1* and *CER3* genes required for the production of waxes by the alkane-forming branch of wax biosynthesis, but not the mutants with lesions in the *CER4* gene required for the production of waxes by the acyl reduction pathway. The distinguishing feature between *cer1* and *cer3* is that *cer1* has slightly increased amounts of aldehydes and reduced primary alcohol levels, whereas *cer3* exhibits a major reduction in aldehydes and similar amounts of primary alcohols to the wild type. Thus, the wax profile of AtARREOX lines is most similar to that of the *cer1* mutant. Besides cuticular wax changes, some AtARREOX lines additionally display reduced plant height and reduced fertility previously described for the *cer1-1* and *cer3-1* mutant alleles (Koornneef et al., 1989), and organ fusion phenotypes characteristic of *cer3*, but not *cer1* mutants (Aarts et al., 1995; Chen et al., 2003; Bourdenx et al., 2011).

### AtARRE Promotes CER1 Degradation by the 26S Proteasome

**Figure 5:**
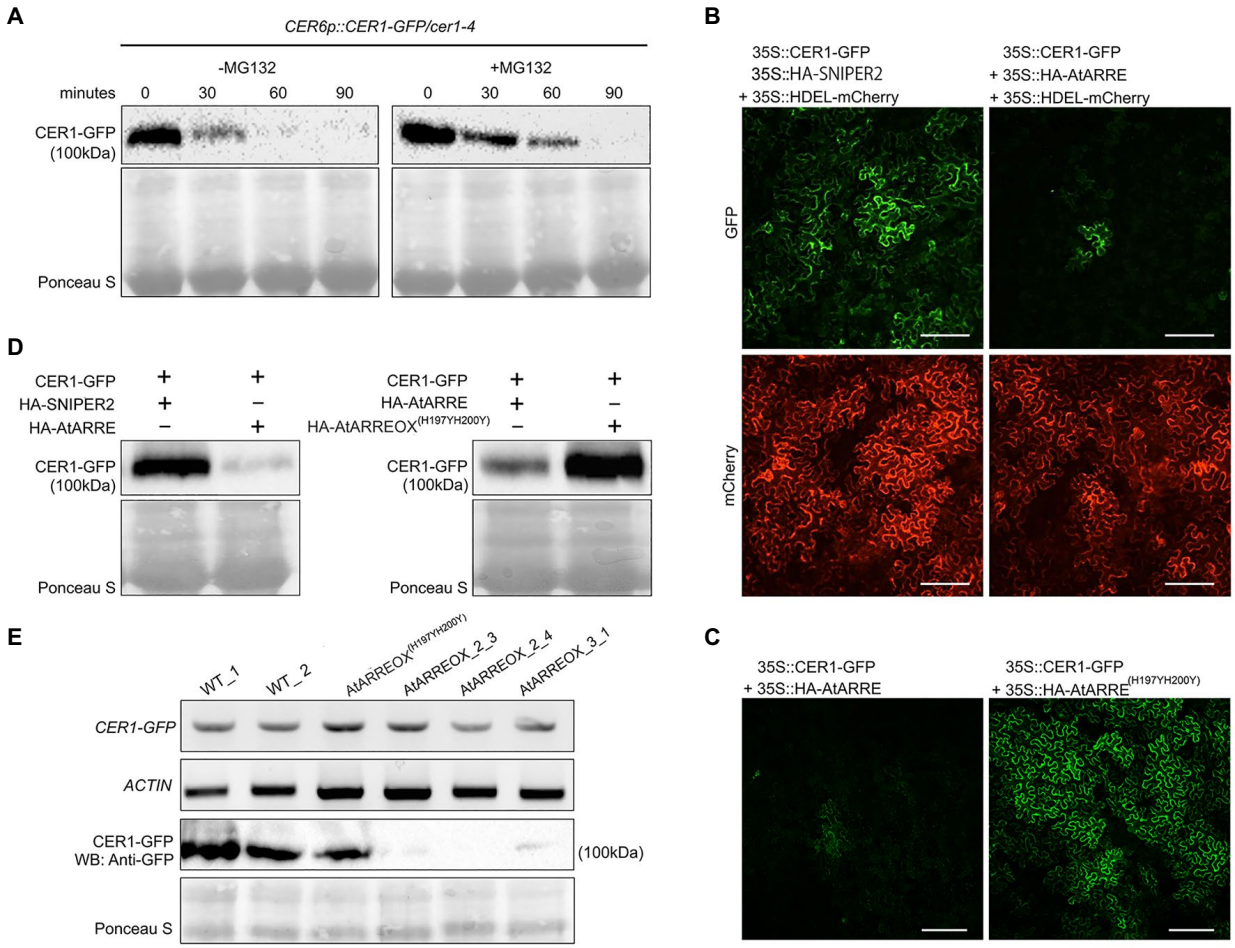
AtARRE promotes CER1 degradation in the *Nicotiana benthamiana* transient expression system and in stable transgenic lines of Arabidopsis. **(A)** CER1 is turned over by the 26S proteasome *in planta*. Total proteins were extracted from 8-day-old *CER6pro:CER1-GFP*/*cer1-4* transgenic plants and incubated with (+) or without (−) 40μM MG132. CER1-GFP protein levels were detected over time by immunoblotting using anti-GFP antibody. **(B,C)** Transient expression in *N. benthamiana* leaf epidermal cells. CER1-GFP and an internal control HDEL-mCherry were co-expressed with HA-SNIPER2 E3 ligase or HA-AtARRE E3 ligase. CER1-GFP was co-expressed with HA-AtARRE or HA-AtARRE^(H197YH200Y)^ with mutated RING domain. GFP fluorescence and mCherry fluorescence were examined by confocal microscopy. Scale bars=100μm. 4 technical replicates and 16 biological replicates have been performed. **(D)** Amounts of CER1-GFP protein were determined by immunoblotting using anti-GFP in the same leaves assayed for fluorescence in (B,C). **(E)** The AtARRE-dependent CER1 degradation in stable transgenic lines of Arabidopsis. RNA was extracted from 4-week-old Arabidopsis leaves. The *CER1-GFP* steady-state transcript levels were determined by RT-PCR, and *ACTIN* was used as an internal control (top two rows). Total protein was extracted from the 4-week-old plant leaves. The CER1-GFP protein level was determined by immunoblotting using anti-GFP antibody; Ponceau S staining shows equal protein loading (bottom two rows).

To determine whether CER1 is subjected to 26S proteasome-dependent degradation, we performed a modified cell-free degradation assay. For this purpose, we made *CER6pro:CER1-GFP/cer1-4* transgenic lines in which the *CER6pro:CER1-GFP* transgene complemented the *cer1-4* wax deficiency (Supplementary Figure S4A). Total proteins extracted from 8-day-old *CER6pro:CER1-GFP/cer1-4* seedlings were incubated with or without the 26S proteasome inhibitor MG132 for 90min and protein levels determined by immunoblotting. The CER1-GFP amounts decreased rapidly in the absence of MG132, but in the presence of MG132, the levels of CER1-GFP remained notably higher over time, suggesting that the 26S proteasome is involved in CER1 proteolysis (Figure 5A).

We next carried out an Agrobacterium-mediated transient expression assay in *N. benthamiana* leaves. Agrobacterium cell cultures expressing *35Spro:CER1-GFP* and *35Spro:HA-AtARRE* were co-infiltrated on one half of a *N. benthamiana* leaf. Cell cultures expressing *35Spro:CER1-GFP* and *35Spro:HA* empty vector or *35Spro:CER1-GFP* and the *35pro:HA-SNIPER2* vector containing the *SNIPER2* E3 ligase gene not involved in wax deposition were co-infiltrated symmetrically on the other half of the same leaf as negative controls (Supplementary Figure S4B). *35Spro:HDEL-mCherry* was also included in each sample as a marker for ER visualization. In *N. benthamiana* cells transformed with CER1-GFP and *35Spro:HA* empty vector, the GFP signal could be easily detected after 48h, persisted past 72h and was undetectable 96h after infiltration (Supplementary Figure S4C). Bright GFP fluorescence was also detected in cells co-expressing CER1-GFP and the SNIPER2 E3 ligase control 3days post-infiltration, but not in those cells co-expressing CER1-GFP and AtARRE (Figure 5B). In contrast, similar intensity of mCherry fluorescence from the ER-localized HDEL-mCherry marker was detected in all infiltrated regions on both sides of the leaf (Figure 5B). Immunoblot analysis confirmed that CER1-GFP protein level was much lower in the presence of AtARRE than in the negative control sample expressing SNIPER2 E3 ligase (Figure 5D). Unlike the native AtARRE protein, co-expression of CER1-GFP and the AtARRE^(H197YH200Y)^ protein with mutated RING domain did not affect CER1 protein levels, indicating that catalytic activity of AtARRE is required for CER1 degradation (Figures 5C,D). When the substrate CER1 was replaced with CER2, a component of VLCFAs elongation machinery (Haslam et al., 2012), GFP tagged CER2 fluorescence signal intensity was found to be indistinguishable in the presence and absence of AtARRE. Immunoblot results were consistent with microscopy data (Supplementary Figures S4D,E) and demonstrate that AtARRE specifically targets CER1 for degradation by the 26S proteasome.

The AtARRE-dependent degradation of CER1 was further verified in stable transgenic lines of Arabidopsis. Plants harboring the CER1-GFP transgene were crossed with the wild type, AtARREOX, and AtARREOX^(H197YH200Y)^ lines, and the abundance of the CER1-GFP in F1 progeny was examined by immunoblotting. Whereas CER1-GFP transcript accumulation was similar in the F1 progeny from all the crosses, the CER1-GFP protein level in the AtARREOX lines was much lower than observed in the wild type and AtARREOX^(H197YH200Y)^ lines (Figure 5E). Collectively, these results confirm that AtARRE promotes the degradation of CER1, suggesting that CER1 is the ubiquitination substrate of AtARRE.

### CER1 Is Ubiquitinated by AtARRE in a Reconstituted *E. coli* System

**Figure 6:**
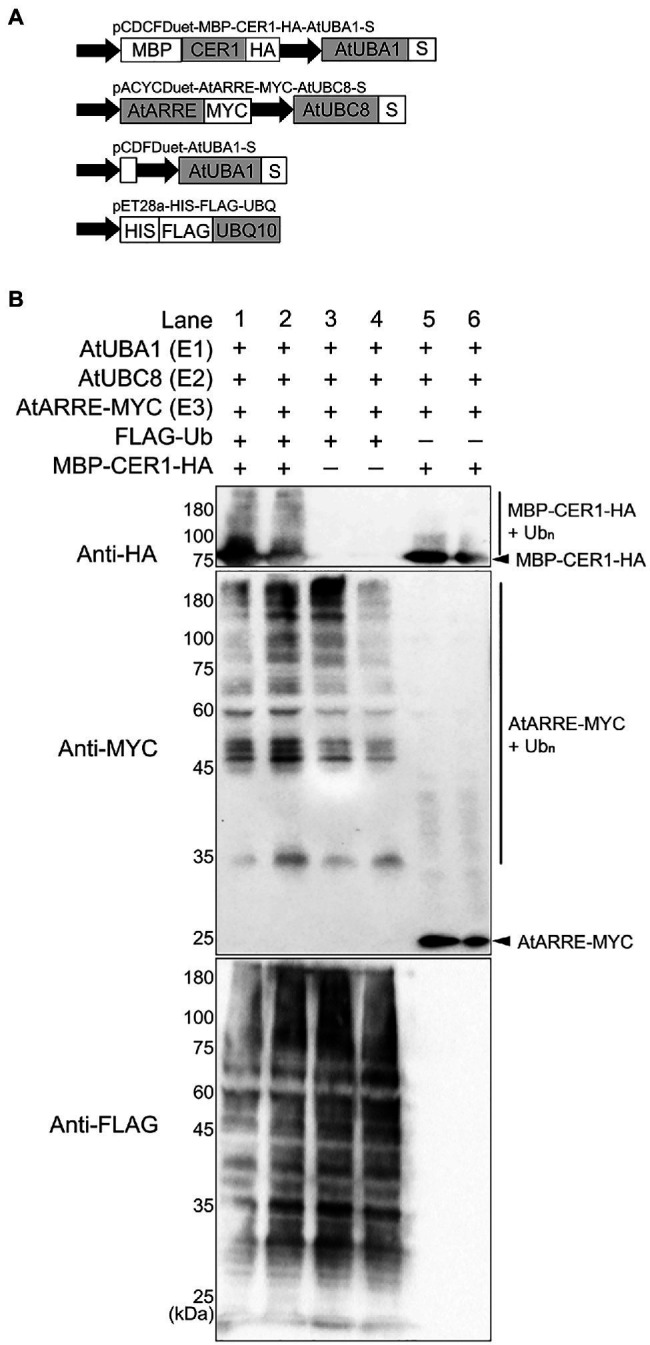
Ubiquitination of CER1 by AtARRE in a heterologous *Escherichia coli* system. **(A)** Schematic representation of the plasmids used in the assay. MBP, maltose-binding protein; HA, hemagglutinin. These constructs were transformed into the *E. coli* Rosetta (DE3) strain to reconstitute the ubiquitination cascade. **(B)** Bacterial lysates from *E. coli* strains expressing (+) or missing (−) combinations of AtUBA1-S (E1), AtUBC8-S (E2), AtARRE-MYC (E3), His-FLAG-UBQ10 (Ub), and MBP-CER1-HA (substrate) (+), and strains missing Ub or CER1 (−) were analyzed by immunoblotting. Anti-HA and anti-MYC antibodies were used to detect ubiquitinated CER1 and self-ubiquitinated AtARRE, respectively. Anti-FLAG antibody was used to detect all Ub conjugates. Two replicates for each combination of constructs are shown.

To directly test whether CER1 is a ubiquitination substrate of the AtARRE E3 ligase, we performed an *in vivo* ubiquitination assay in a heterologous *E. coli* system that expresses the Arabidopsis ubiquitination cascade (Han et al., 2017). In this experiment, recombinant ubiquitination components E1 (AtUBA1-S), E2 (AtUBC8-S), E3 (AtARRE-Myc), ubiquitin (His-Flag-AtUBQ10), and the presumed ubiquitination substrate (MBP-CER1-HA) were co-expressed in *E. coli* (Figure 6A), and bacterial lysates were analyzed by immunoblotting. Our results show that in the presence of all ubiquitination components, a smear indicative of CER1 ubiquitination can be detected by an anti-HA antibody (Figure 6B). Using an anti-Myc antibody, AtARRE-Myc recombinant protein also shows a laddering pattern indicative of AtARRE self-ubiquitination. These data support the *in vitro* ubiquitination assay results that AtARRE is an active E3 ligase and demonstrate that it can ubiquitinate CER1 *in vivo*.

### AtARRE Promotes CER3-GFP Degradation

**Figure 7:**
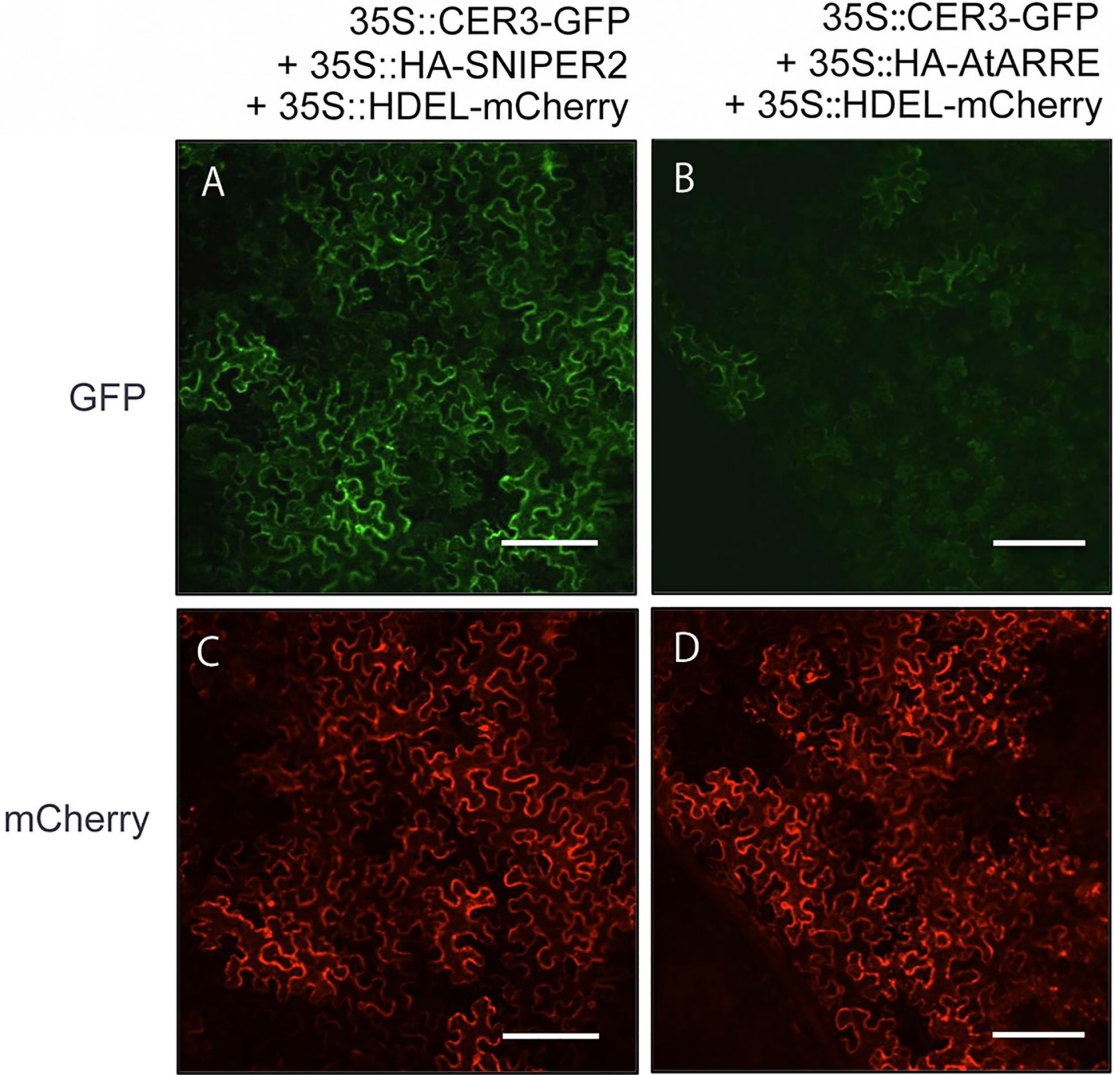
AtARRE promotes CER3 degradation in the *N. benthamiana* transient expression system. **(A,C)** *35Spro:CER3-GFP* was co-expressed with *35Spro:HA-SNIPER2* or **(B,D)** *35Spro:HA-AtARRE. 35Spro:HDEL-mCherry* was included as an internal control and ER marker. GFP fluorescence **(A,B)** and mCherry fluorescence **(C,D)** signals of leaf epidermal cells were examined by confocal microscopy 72h post-infiltration. 12 biological replicates were performed. Scale bars=100μm.

Even though stem wax profiles of AtARRE overexpressors are more similar to the *cer1* than the *cer3* mutant, the overexpression lines also exhibit organ fusions previously detected in *cer3*, but not in *cer1* mutants. Because CER1 and CER3 proteins are highly related and share 35% sequence identity (Bernard et al., 2012), it is tempting to speculate that in addition to CER1, CER3 may also be a target of the AtARRE-mediated ubiquitination and UPS proteolysis resulting in the dual *cer1-* and *cer3*-like phenotypic features of AtARRE overexpression lines. To investigate whether this is the case, we tested whether AtARRE affects the protein levels of CER3-GFP in *N. benthamiana* leaf epidermal cells. Agrobacterium cells expressing *35Spro:CER3-GFP*, the *35Spro:HA-AtARRE*, and the *35Spro:HDEL-mCherry* transgenes were co-infiltrated on one half of a *N. benthamiana* leaf, while Agrobacterium cells harboring *35Spro:CER3-GFP*, *35Spro:HA-SNIPER2*, and *35Spro:HDEL-mCherry* were co-infiltrated on the other side of the same leaf as a negative control. Similar to CER1-GFP, AtARRE promoted CER3-GFP degradation as indicated by considerably reduced CER3-GFP fluorescence in the presence of transiently co-expressed AtARRE in comparison with the control co-expressing CER3-GFP with HA-SNIPER2 E3 ligase. By contrast, the signal of the internal control HDEL-mCherry was prominent and indistinguishable on both halves of the leaf (Figure 7). These results demonstrate that CER3 is likely also the AtARRE ubiquitination target. We tried to confirm that AtARRE controls the levels of CER3 in transgenic lines of Arabidopsis, but consistently failed to detect CER3-GFP protein on immunoblots.

### *AtARRE* Gene Is Expressed in Tissues That Exhibit Low Wax Production and Upon Exposure to Pathogens

**Figure 8:**
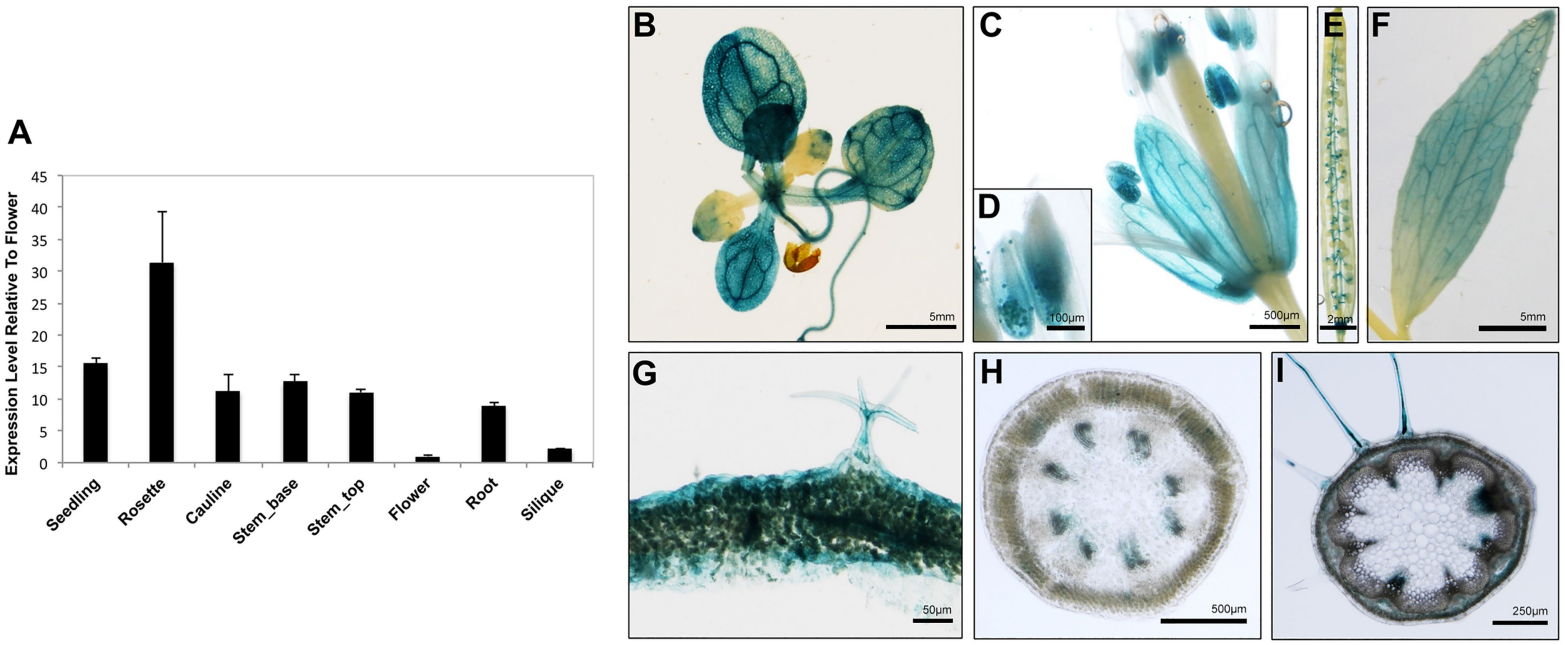
Expression pattern of *AtARRE* gene in different plant tissues and cell types. **(A)** qRT-PCR analysis of *AtARRE* gene expression in the WT relative to flowers and normalized to *ACTIN*. Seedling and root tissue samples were derived from 10-day-old WT seedlings, and all other aerial tissues samples were derived from 6-week-old WT. Error bars represent means±SD (*n*=4). **(B–I)** Putative *AtARRE* promoter activity determined by GUS assay in transgenic lines expressing the *AtARREpro:GUS* reporter. Promoter activity was examined after GUS staining in 14-day-old seedlings **(B)**, flowers **(C)**, anthers containing pollen grains **(D)**, siliques **(E)**, cauline leaves **(F)**, cross section of rosette leaf from a 6-week-old plant **(G)**, cross section of the top 3cm **(H)** and the bottom 3cm **(I)** of the inflorescence stem of a 6-week-old plant.

To obtain clues regarding the functional significance of the AtARRE-mediated protein degradation of wax biosynthetic enzymes, we investigated the transcription profile of *AtARRE* gene in different plant tissues using qRT-PCR (Figure 8A). The *AtARRE* gene was expressed in all tissues examined, with higher expression in rosette leaves and lower expression in flowers and siliques. To further determine the *AtARRE* expression pattern in different cell types and at different Arabidopsis developmental stages, an 808bp fragment of genomic sequence immediately upstream of the *AtARRE* coding region was fused to the β-glucuronidase (GUS) reporter gene, and this *AtARREpro:GUS* reporter was transformed into wild-type Arabidopsis. GUS activity was examined in tissue samples from mature plants and in developing seedlings of ten independent transgenic lines.

In mature plants, high GUS activity was detected in the fully expanded rosette leaves, sepals, pollen grains, and cauline leaves (Figures 8B–F). These results are consistent with the published RNA-seq data showing high *AtARRE* expression in mature leaves and sepals (Klepikova et al., 2016). Strong GUS signal was also detected in, but not specific to, the epidermal cells of rosette leaves, as well as trichomes, which are specialized epidermal cells (Figure 8G). Surprisingly, no expression of *AtARRE* was detected in the epidermal cell layer of the stem (Figures 8H,I). This may be due to the fact that the 5′ promoter fragment used in the *AtARREpro:GUS* construct does not contain the regulatory element required for the *AtARRE* expression in stem epidermal cell layer. In fact, according to published RNA-seq and microarray data, *AtARRE* shows the highest expression level in the senescent first internode of the stem, and higher expression was detected in the epidermal cell layer at the bottom of the inflorescence stem compared to the top of the stem (Suh et al., 2005; Klepikova et al., 2016). During seedling development, GUS activity was first detected in roots in 3-day-old seedlings and cotyledons at 4days after imbibition (Supplementary Figure S5), but the GUS staining was much more pronounced in both organs in 5-day-old seedlings.

The observed expression profile fits the role of AtARRE as a negative regulator of wax biosynthesis in tissues that exhibit no or low wax production, such as mature cotyledons in older developing seedlings, as well as fully expanded rosette leaves and older internodes at the bottom of the stem in mature plants. Unexpectedly, *AtARRE* was also found to be expressed in roots, even though *CER1* and *CER3* genes encoding AtARRE ubiquitination substrates are not expressed in roots or are expressed at a very low level, respectively (Bourdenx et al., 2011).

**Figure 9:**
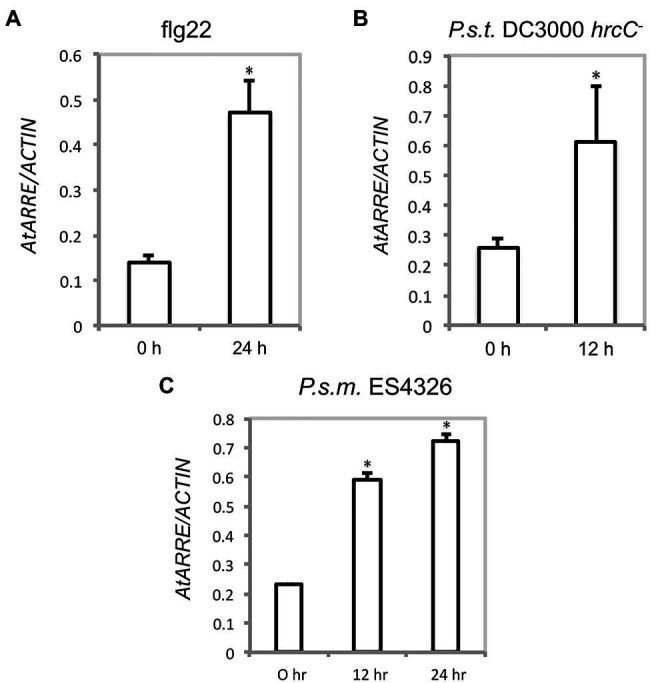
*AtARRE* gene expression is induced by flg22 and pathogen treatment. **(A)** Four-week-old WT plants were infiltrated with 100nM flg22, **(B)** *P.s.t*. DC3000 *hrcC^−^* at a dose of OD_600_=0.002, or **(C)** *P.s.m*. ES4326 at a dose of OD_600_=0.0002. Samples were collected 0, 12, or 24h after treatment. Expression level of *AtARRE* was measured by qRT-PCR, and values were normalized to the level of *ACTIN*. Error bars represent means±SD (*n*=4). Significant difference compared with WT was determined by Student’s *t* test and indicated by an asterisk (*p*<0.01).

Because AtARRE was discovered in a reverse genetic screen conducted to identify novel plant immunity-related E3 ligases, we were interested in determining whether AtARRE is induced upon exposure to pathogens. An earlier study has shown that *AtARRE* is upregulated after treatment with chitin, a potent elicitor of plant defense responses (Libault et al., 2007). Our qRT-PCR analysis revealed that elicitation with flg22, a peptide derived from bacterial flagellin, also results in a major increase in *AtARRE* expression (Figure 9A). In addition, infiltration of plants with the type III secretion deficient bacterial strain *Pseudomonas syringae* pv. *tomato* (*P.s.t*.) DC3000 *hrcC^−^* and the virulent bacterial strain *P. syringae* pv. *maculicola* (*P.s.m*.) ES4326 (Figures 9B,C) strongly upregulated *AtARRE* expression. Collectively, these data suggest that AtARRE may be involved in regulating cuticular wax biosynthesis in response to pathogen attack.

## Discussion

As an integral part of the cuticle, wax protects plants against diverse biotic and abiotic stress factors present in their environment. To fulfill this protective role, wax composition and wax load need to be continuously adjusted in response to changing environmental conditions (Shepherd and Wynne Griffiths, 2006; Bernard and Joubès, 2013). This is accomplished by extensive transcriptional, post-transcriptional, and post-translational regulation of cuticular wax metabolism (Lee and Suh, 2015a).

VLC alkanes are the major cuticular wax component in many plant species including Arabidopsis, where they comprise more than 70% of the total wax amount in leaves and 50% in stems (Bourdenx et al., 2011). VLC alkane production is catalyzed by CER1 and CER3 enzymes, which together with the cytochrome B5 form a multiprotein ER-membrane-associated complex (Bourdenx et al., 2011; Bernard et al., 2012) Not surprisingly, both enzymes act as key control points for wax biosynthesis. Several transcription factors including MYB30, MYB94, MYB96, DEWAX, and DEWAX2 regulate the expression of *CER1* and/or *CER3* in specific organs of Arabidopsis or in response to environmental stress (Raffaele et al., 2008; Seo et al., 2011; Go et al., 2014; Lee and Suh, 2015b). Components involved in chromatin remodeling are also required for the transcriptional regulation of *CER1* or *CER3*. HISTONE MONOUBIQUITINATION 1 and 2 (HUB1 and HUB2) are two E3 ligases that are involved in histone monoubiquitination and active chromatin formation, which leads to the transcriptional activation of wax biosynthetic genes, such as *CER1* (Ménard et al., 2014). GENERAL CONTROL NON-REPRESSED PROTEIN5 (GCN5) is a histone acetyltransferase that positively modulates *CER3* expression *via* histone acetylation (Wang et al., 2018c). In addition to transcriptional regulation, two classes of small RNAs, tasiRNA and miRNA, post-transcriptionally control *CER3* and *CER1* transcript levels, respectively (Lam et al., 2012, 2015; Li et al., 2019). Finally, studies on the E3 ligase MIEL1 have shown that it negatively regulates wax accumulation in aerial plant organs (Gil et al., 2017). MIEL1 controls the stability of wax-associated transcription factors MYB96 and MYB30 and thereby indirectly affects the expression of their downstream targets *CER1* and *CER3* (Marino et al., 2013; Lee and Seo, 2016; Gil et al., 2017). Even though the regulatory framework governing wax accumulation has been established, the intricacies of this process remain poorly understood.

Here, we demonstrate that CER1 and CER3 protein levels, and thus alkane formation, are also controlled directly by the AtARRE RING-type E3 ubiquitin ligase that we identified in our SNIPER genetic screen (Tong et al., 2017). We found that AtARRE overexpression in wild-type Arabidopsis results in phenotypes characteristic of cuticular wax deficiency, including glossy stems and siliques, reduced fertility and fusions between aerial organs, suggesting that AtARRE is a negative regulator of wax biosynthesis (Figures 1A, 2). The wax analysis of AtARRE overexpression lines confirmed that they have reduced stem and leaf wax loads (Figures 1B–F) and revealed that their wax profile most closely resembled that of the *cer1* mutant (Figure 4). Functional characterization of AtARRE showed that this protein has E3 ubiquitin ligase activity and that this activity depends on the integrity of the key amino acid residues in its RING domain. When these conserved residues in the RING domain were mutated, the AtARRE protein no longer had any effect on the stem wax accumulation when it was overexpressed in Arabidopsis (Figure 3). These results demonstrate that E3 ligase activity of AtARRE is essential for its function in wax biosynthesis.

Because wax composition of the AtARRE overexpressors was most similar to the *cer1* mutant, we tested whether CER1 was the AtARRE ubiquitination substrate. An *in vivo* ubiquitination assay in a heterologous *E. coli* system expressing the Arabidopsis ubiquitination cascade confirmed AtARRE-mediated CER1 ubiquitination (Figure 6). Furthermore, transient co-expression of AtARRE and CER1 in both *N. benthamiana* leaves and in stable transgenic lines of Arabidopsis resulted in AtARRE-dependent degradation of CER1. Thus, AtARRE E3 ligase negatively regulates cuticular wax accumulation by ubiquitinating VLC alkane biosynthetic enzyme CER1 and targeting it for degradation by the 26S proteasome (Figure 5).

It is well-established that the UPS is redundant and that individual proteins may be targeted by multiple E3 ligases. Conversely, a single E3 ligase may have the ability to target multiple substrates for degradation (Iconomou and Saunders, 2016). CER1 and CER3, two key VLC alkane biosynthetic enzymes, share 35% amino acid identity, and both are integral membrane proteins with eight conserved His clusters at their N-terminus and an uncharacterized WAX2 domain at their C-terminus (Bernard et al., 2012). Due to their sequence similarity, it was possible that both of these proteins were substrates of the AtARRE E3 ligase. This fact, together with the observation that AtARRE overexpressors exhibit organ fusions similar to those detected in *cer3*, but not in *cer1* mutants, prompted us to investigate whether AtARRE additionally controls CER3 levels. As previously demonstrated for CER1, AtARRE co-expression with CER3 in the *N. benthamiana* leaf indeed stimulated CER3 degradation (Figure 7). Thus, CER3 is also likely an AtARRE ubiquitination substrate.

The expression analysis of *AtARRE* gene revealed that it is preferentially expressed in tissues that exhibit no or low wax production such as roots and cotyledons in the later stages of seedling development, as well as older rosette leaves and inflorescence stem sections (Figure 8). These data suggest that the primary role of AtARRE may be to target CER1 and CER3 proteins for degradation in order to terminate wax production *via* the alkane pathway in tissues where it is no longer needed. Additionally, *AtARRE* gene transcription is upregulated upon exposure to bacterial pathogen *P. syringae* (Figure 9). Previous analysis of the *cer1* mutant showed that reduced VLC alkane levels in cuticular wax are associated with increased cuticle permeability, but have no major effect on plant immunity. In contrast, *CER1* overexpression resulted in alkane overproduction and decreased cuticle permeability, but surprisingly also in greater susceptibility to *P. syringae* (Bourdenx et al., 2011). Similarly, cucumber (*Cucumis sativus*) lines overexpressing *CER3* exhibited enhanced susceptibility to the fungal pathogen *Botrytis cinerea* (Wang et al., 2015). Thus, induction of *AtARRE* upon exposure to pathogens and the resulting degradation of CER1 and CER3 may serve as a regulatory mechanism aimed at decreasing VLC alkane levels and optimizing cuticular wax composition to enhance plant resistance to bacterial pathogens. However, further work is needed to clearly define the role of AtARRE in plant immunity.

Recently, *AtARRE* was also reported to be induced by sodium chloride and ABA treatments (Wang et al., 2018a). Analyses of seed germination, stomatal closure, root elongation, and expression of ABA-responsive genes in *atarre* mutants showed that all these processes were hypersensitive to ABA, whereas AtARRE overexpressors exhibited reduced ABA sensitivity, leading the authors to propose that AtARRE is a negative regulator of ABA-dependent abiotic stress responses in plants (Wang et al., 2018a). This study did not identify the AtARRE ubiquitination target or propose the molecular mechanism governing these ABA stress responses, but it is unlikely that the AtARRE-mediated CER1 degradation by the proteasome that controls attenuation of cuticular wax biosynthesis in the shoot described here is involved. On the contrary, water deficit, salt, and ABA treatment have been shown to cause a large increase in wax amount in the leaf cuticle, predominantly due to an increase in VLC alkane content (Kosma et al., 2009). A huge induction of the *CER1* alkane biosynthetic gene consistent with the elevated alkane amounts was also observed, presumably resulting in greater CER1 protein levels. Thus, even though the ABA signaling would not be expected to rely on the AtARRE-CER1 module, it is conceivable that AtARRE function in ABA-dependent signal transduction requires destruction of a different and as yet undiscovered protein target. A good example of an E3 ligase with multiple physiological roles that depend on proteasomal degradation of different protein substrates is MIEL1. MIEL1 controls seed germination and cuticular wax accumulation in Arabidopsis stems by primarily targeting MYB96 transcription factor for degradation, but in addition attenuates pathogen defense responses by promoting degradation of MYB30 (Marino et al., 2013; Lee and Seo, 2016).

## Conclusion

Our findings indicate that AtARRE E3 ubiquitin ligase negatively regulates cuticular wax accumulation in Arabidopsis shoots by promoting degradation of CER1 and CER3 VLC alkane biosynthetic enzymes. Based on its expression in mature and senescing tissues and its induction upon plant exposure to pathogens, we propose that AtARRE serves as an efficient regulatory switch that terminates wax biosynthesis *via* the alkane-forming pathway when it is no longer required, or to optimize wax composition in response to pathogen infection.

## Data Availability Statement

The raw data supporting the conclusions of this article will be made available by the authors, without undue reservation.

## Author Contributions

LK and XL planned and designed research. SL, MT, and LZ performed the experiments. SL, LZ, XL, and LK analyzed and discussed the data. SL and LK wrote the manuscript. All authors contributed to the article and approved the submitted version.

## Funding

This work was supported by Natural Sciences and Engineering Research Council of Canada Discovery Grants to XL and LK, and scholarships from the China Scholarship Council to SL and MT.

## Conflict of Interest

The authors declare that the research was conducted in the absence of any commercial or financial relationships that could be construed as a potential conflict of interest.

## Publisher’s Note

All claims expressed in this article are solely those of the authors and do not necessarily represent those of their affiliated organizations, or those of the publisher, the editors and the reviewers. Any product that may be evaluated in this article, or claim that may be made by its manufacturer, is not guaranteed or endorsed by the publisher.

## Footnotes

^1^ arabidopsis.org/
