# Supplementary material for: The ARRE RING-Type E3 Ubiquitin Ligase Negatively Regulates Cuticular Wax Biosynthesis in Arabidopsis thaliana by Controlling ECERIFERUM1 and ECERIFERUM3 Protein Levels
Source: Front Plant Sci. 2021 Oct 26;12:752309. doi: 10.3389/fpls.2021.752309 (PMC8576476; doi:10.3389/fpls.2021.752309)
Supplement: Supplementary file 1 [file Data_Sheet_1.PDF]

Supplementary Material

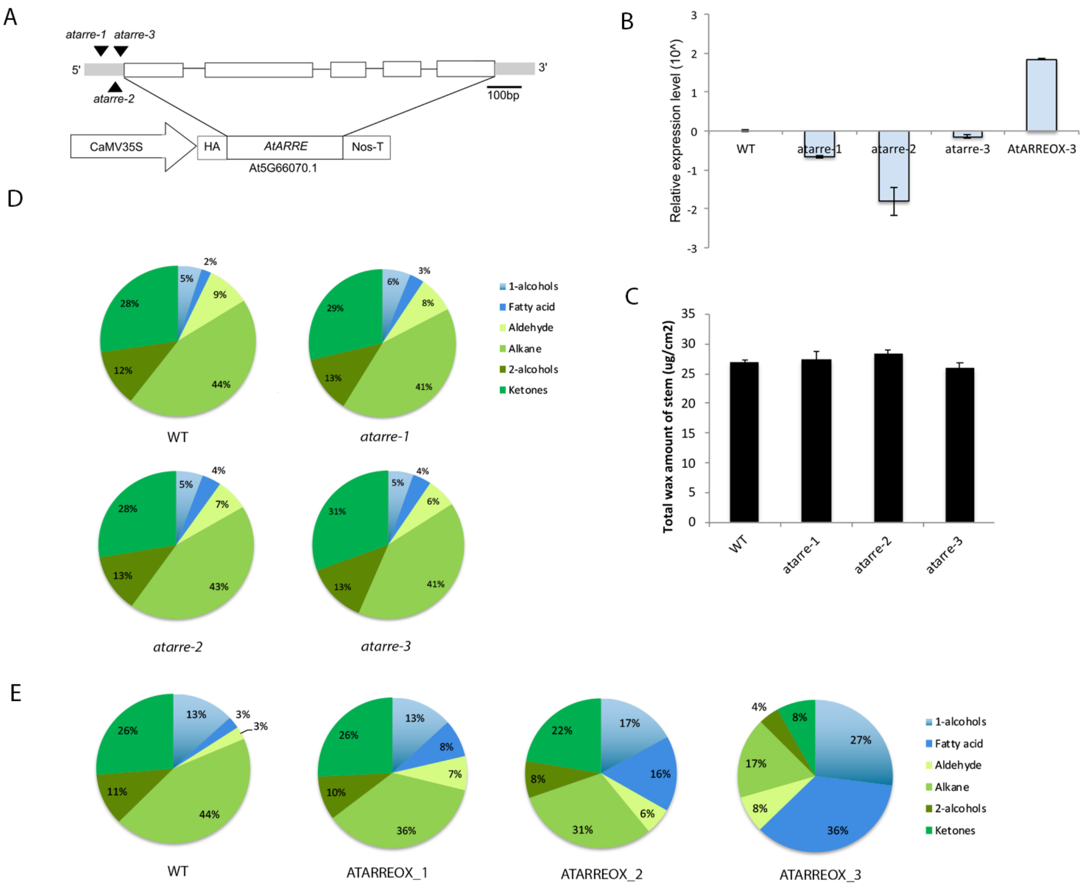

Supplementary Figure 1. Analysis of AtARREOX lines and *atarre* mutants.

(A) Schematic diagram of T-DNA insertion locations in *atarre* mutants and the original construct for generating AtARREOX lines. **(B)** *AtARRE* gene expression level in stems of the WT, *atarre* mutants, and a representative AtARREOX line, determined by qPCR. *ACTIN* was used as an internal control. The y-axis is shown in the logarithmic scale. Student's *t*-test was applied to identify statistically significant differences ($p<0.05$). Error bars represent means ± SD (n=3). **(C)** Stem wax loads of *atarre* T-DNA mutants compared to the WT. **(D,E)** Stem wax composition of *atarre* mutant alleles (D) and AtARREOX lines (E) compared to the WT. Alkane pathway-derived compounds were labelled in shades of green color. Primary alcohol and fatty acids are labelled in shades of blue color.

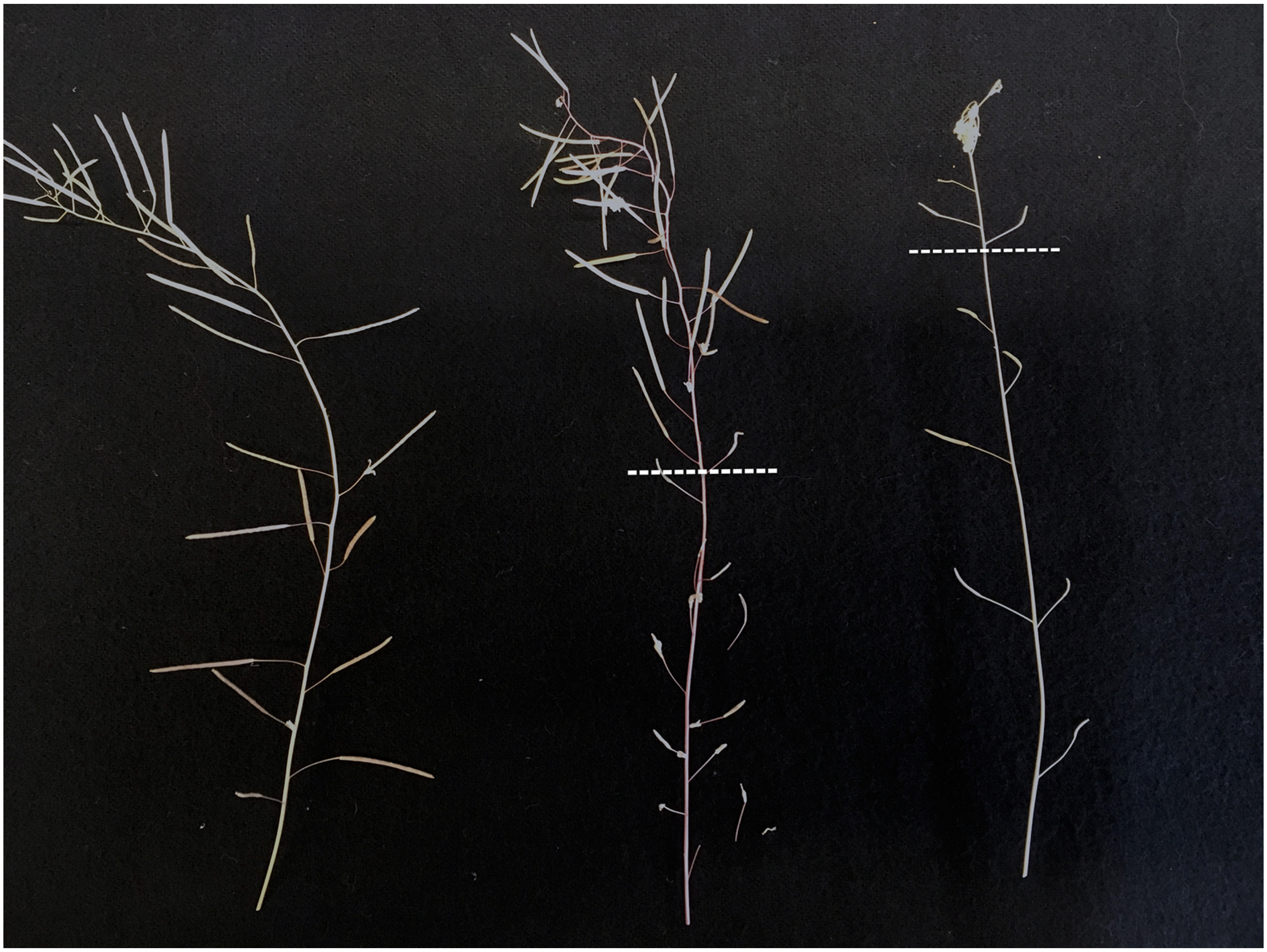

Supplementary Figure 2. Fertility of AtARREOX lines can be restored by high humidity.

Dry stems of WT Col-0 (left), AtARREOX_2 (middle), and AtARREOX_3 (right). WT was grown under normal humidity conditions; AtARREOX lines were grown under normal (below dashed line) and high-humidity conditions (above dashed line). High-humidity conditions were created by covering the plant with a plastic bag. AtARREOX_3 line showed a severe organ fusion phenotype and fertility could not be fully recovered by growth in high humidity.

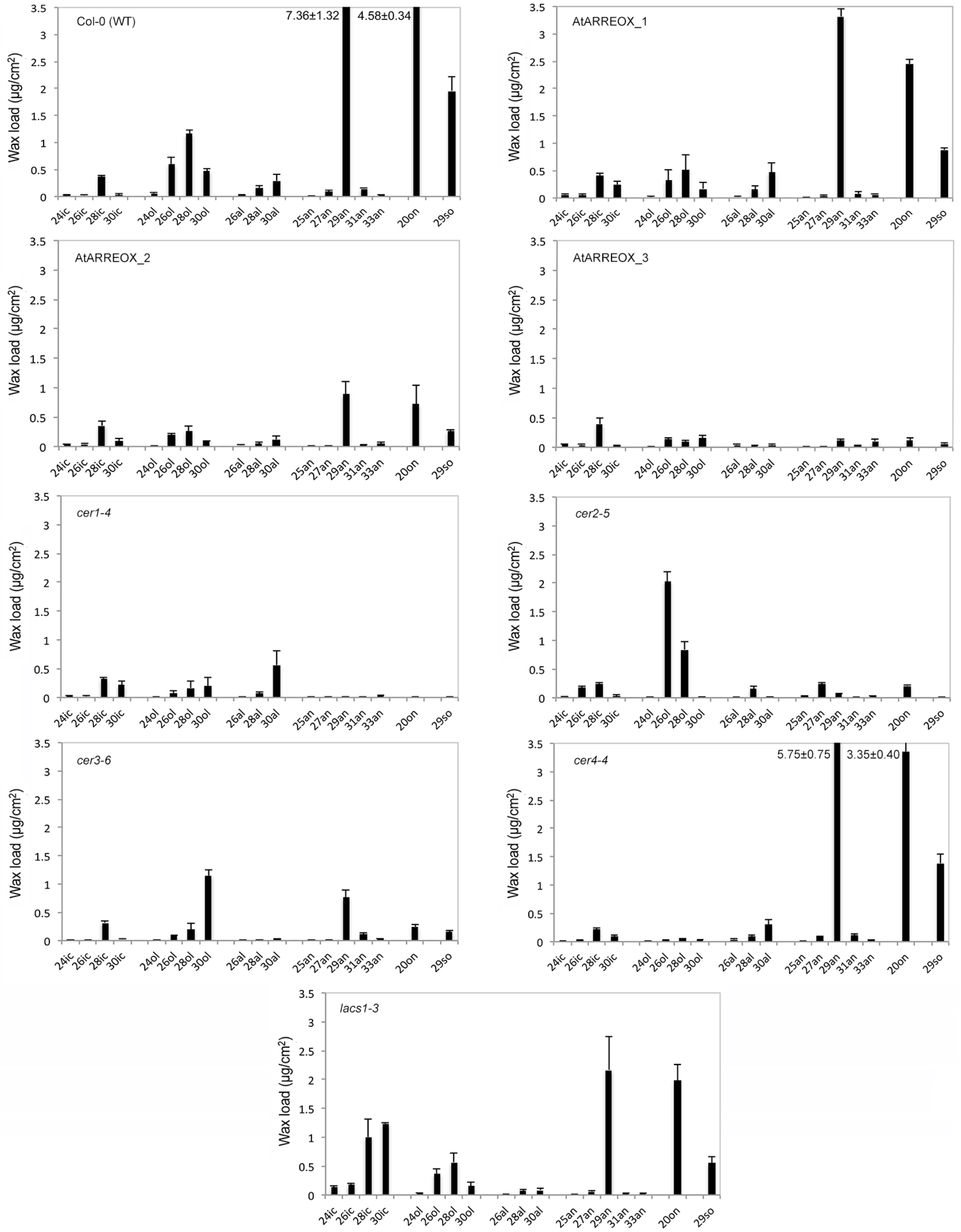

Supplementary Figure 3. Stem wax load and composition of WT Col-0, AtARREOX lines, *cer1-4*, *cer2-5*, *cer3-6*, *cer4-4*, and *lacs1-3* mutants.

Total stem wax load of 6-week-old plants were determined by GC-FID. Values are means of four biological replicates and error bars represent SD.

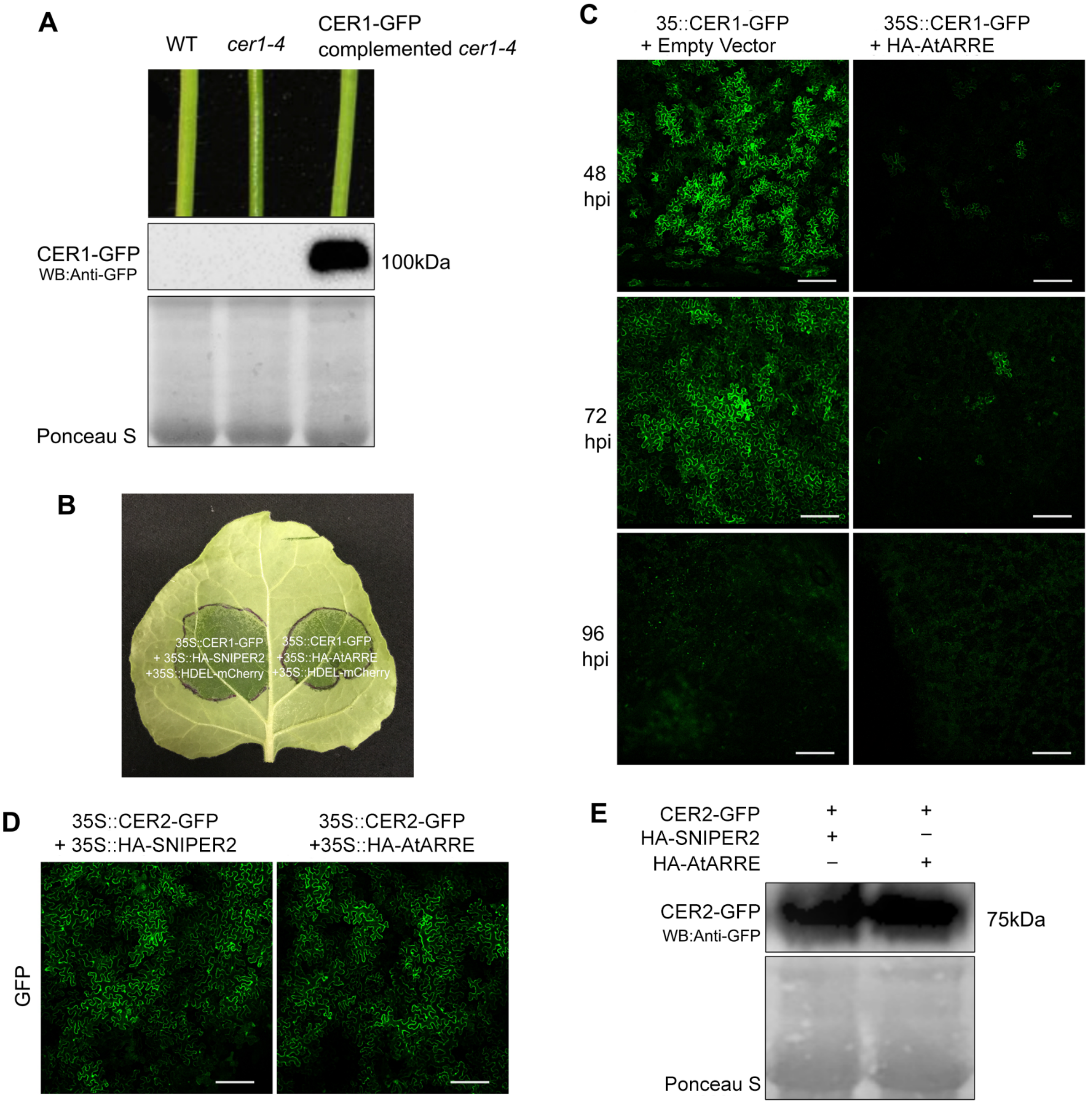

Supplementary Figure 4. AtARRE specifically targets CER1 for degradation in *N. benthamiana* leaves.

(A) Stems of the WT, *cer1-4*, and *cer1-4* complemented with *CER6pro:CER1-GFP* transgene (top). CER1-GFP protein level examined by immunoblotting using anti-GFP antibody (middle). Ponceau S staining indicates equal protein loading (bottom). **(B)** An infiltrated *N. benthamiana* leaf showing the combination of constructs used for co-infiltration. **(C)** Leaf samples were collected at the indicated time after infiltration and the GFP fluorescence for each sample was examined by confocal microscopy. hpi, hours post infiltration. *35Spro:CER1-GFP* was co-expressed with a *35Spro:HA* empty vector (left panels) or *35Spro:HA-AtARRE* (right panels) **(D)** *35Spro:CER2-GFP* was co-expressed with *35Spro:HA-SNIPER2* (left) or *35S:HA-AtARRE* (right) and GFP fluorescence was examined by confocal microscopy. Scale bars = 100μm. **(E)** CER2-GFP protein levels from the protein extracts derived from D were determined by immunoblotting using anti-GFP antibody. Ponceau S staining indicates equal protein loading.

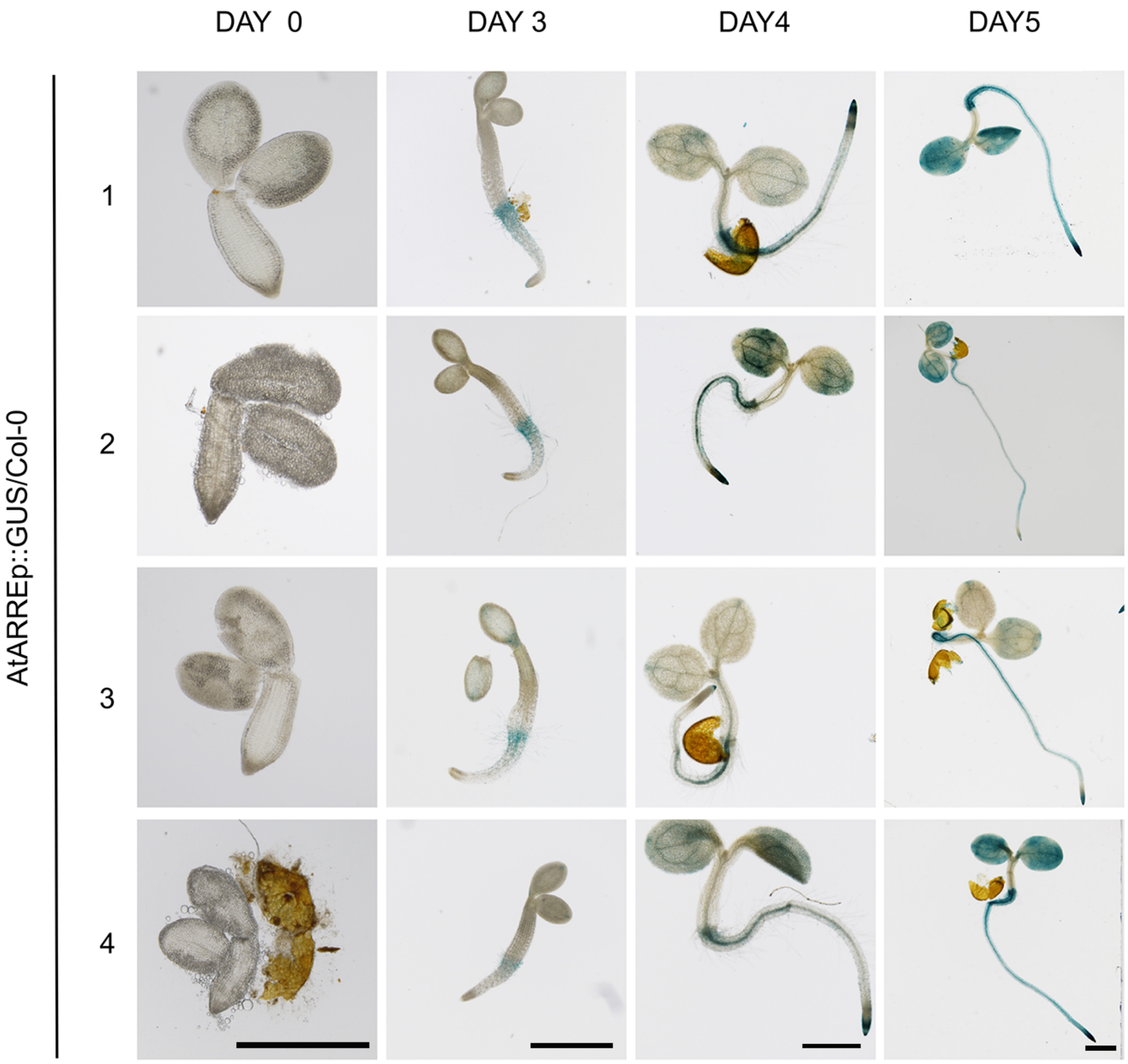

Supplementary Figure 5. Cell-type specific expression of *the AtARRE* gene determined by GUS activity assays in transgenic lines expressing *AtARREpro:GUS* construct.

Tissue samples from ten independent transgenic lines were examined for GUS activity, with four of these lines analyzed in detail. Seedlings of four *AtARREpro:GUS* transgenic lines were harvested on the day indicated and stained in GUS staining solution. Columns indicate days after imbibition, with day 0 representing embryos from dry seed. Scale bar = 1mm.

Supplementary Table 1. Primers used in this study.

Primer	Sequences (5'-3')
**Primers used for genotyping T-DNA lines of mutants**	
atarre-1 LP	CTGACTCTGGACGGCACTTAG
atarre-1 RP	GTACAGGGAAAGGTGAGGAGG
atarre-2 LP	AAGGCATCCAATTCCAGACTC
atarre-2 RP	GTACAGGGAAAGGTGAGGAGG
atarre-3 LP	TGAGAAGTTGGGTGGTGTTTC
atarre-3 RP	CAAACCTGAAGGCAGACAGAG
**Primers used for genotyping transgenic lines of AtARREOX**	
TransAtARREOX_F	TGTTCCAGACTACGCTGTCG
TransAtARREOX_R	TCCTCTGATGAAACCGCTCT
**Primers used for RT-PCR & qPCR**	
ACT1-NF	CTCAGTACCTTCCAGCAGATGT
ACT1-NR	AAAAACCCGGCTTGAGAAAT
ATARRE_RT-PCR_F	TCTTGCTCTGTCTGCCTTCA
ATARRE_RT-PCR_R	GCACAAGGGACAAGAAGCAT
CER1F-RT	AGTAGATTAGCAGCAGCTGTTG
CER1R-RT	GCTCTTCTCTTGTTGTTCCTT
CER3-qPCR-F	CTCATCTCCTGTTCCACATCC
CER3-qPCR-R	TCAATGGAACACCAGCTACG
**Primers used for site-directed mutagenesis of ATARREOX lines**	
H197200Y_F	TATATGTTCTACCTACCATGCATCGAC
H197200Y_R	GGTAGGTAGAACATATAATGGCAGTGCGG
ATARREmutate_Seq	AGGCTTGTTCGTGAGCGTAT
**Primer used for *CER3* transient expression in tobacco leaves**	
CER3cDNA_attbF	GGGGACAAGTTTGTACAAAAAAGCAGGCTCAATGGTT GCTTTTTTATCAGCTTGG
CER3cDNA_attbR_WSTOP	GGGGACCACTTTGTACAAGAAAGCTGGGTCTCAATTTG TGAGTGAAGAAACAGCA
CER3cDNA_attbR_NoSTOP	GGGGACCACTTTGTACAAGAAAGCTGGGTCATTTGTGA GTGAAGAAACAGCA
**Primers used for GUS assay**	
LP_attb1_AtARRE	GGGGACAAGTTTGTACAAAAAAGCAGGCTTAGAGGGA TGATTGAGGTAAGTTTAAG
RP1_attb2_ AtARRE	GGGGACCACTTTGTACAAGAAAGCTGGGTTATCTGCAA AAGAGATGAAGAGGAGG
RP2_attb2_ AtARRE	GGGGACCACTTTGTACAAGAAAGCTGGGTTAAGATGT CTTCTGCACAAGGGACAA
GUS_Rseq	CTGTGGAATTGATCAGCGTTGG

Primers used for AtARRE and CER1 expression in bacteria	
ATARRETMdel_F_EcoRI _28b	GACTGAATTCGAGGCTTGTTCGTGAGC
ATARRETMdel_R_SalI	CTAGTCGACAAGATGTCTTCTGCACAAG
T7terminator_R_Seq	CTCAAGACCCGTTTAGAGGC
ATARRETMdel_BamHI	GAAGGATCCAGGCTTGTTCGTGAG
ATARRETMdel_StuI	GTCAGGCCTAAGATGTCTTCTGCACAAG
CER1TMdel_EcoRI_F	GACTTGAATTCCTCTTTGTCGCTGAG
